# CD47 antibody-armed oncolytic adenovirus promotes chimeric antigen receptor macrophage phagocytosis and antitumor immunity

**DOI:** 10.1186/s40164-025-00696-7

**Published:** 2025-08-14

**Authors:** Zhongbing Qi, Shichuan Hu, Jing Zhao, Xianglin Xu, Anliang Huang, Yu Qin, Yao Zhang, Qingzhe Yang, Jianchuan Hu, Chao Su, Ping Cheng

**Affiliations:** 1 Department of Biotherapy，Cancer Center and State Key Laboratory of Biotherapy，West China Hospital, Sichuan University, 17 People’s South Road, Chengdu, 610041 PR China; 2 Department of Pathology, Chengdu Fifth People’s Hospital, Chengdu, PR China; 3 Cancer Center, Department of Biotherapy，Laboratory of Integrative Medicine, Clinical Research Center for Breast and State Key Laboratory of Biotherapy，West China Hospital, Sichuan University, Chengdu, Sichuan 610041 China

**Keywords:** Macrophages, Chimeric antigen receptor, CD47, Neoantigen-specific T cell response, Tumor immune microenvironment, Immunotherapy

## Abstract

**Background:**

Chimeric antigen receptor (CAR)-T cell therapy has shown success in hematologic malignancies but has encountered challenges in solid tumors. Macrophages, being a potentially effective therapeutic target, have led to the development of several therapeutic strategies due to their unique phagocytic function. This study aimed to develop an effective solid tumor immunotherapy strategy by combining CAR macrophages (CAR-Ms) targeting PD-L1 with CD47 antibody-armed oncolytic adenovirus (oAd-CD47).

**Methods:**

In this study, an adenoviral vector was employed to construct CAR-Ms that target PD-L1 and express IFN-γ. The phagocytic capacity and phenotype of CAR-Ms were tested in vitro. Two mouse tumor models with different immunogenicity were utilized to investigate the anti-tumor efficacy of CAR-Ms in vivo. Subsequently, the synergistic anti-tumor effects of CAR-M and oAd-CD47 and their underlying mechanisms were explored.

**Results:**

CAR-Ms exhibited enhanced phagocytic capacity and proinflammatory (M1) phenotype. These CAR-Ms significantly reduced tumor burden and extended overall survival in mice bearing CT26 colon cancer, a model characterized by high immunogenicity. Compared with CAR-Ms and oAd-CD47 monotherapy, this combination therapy (C + o) achieved superior antitumor efficacy in the CT26 and B16 melanoma mouse models, as well as in the ID8 peritoneal metastasis model. Notably, C + o treatment enhanced tumor-associated macrophage (TAM) phagocytosis and reduced the population of inhibitory immune cell subsets, thereby resulting in enhanced adaptive antitumor T-cell and neoantigen-specific T-cell immunity. Additionally, the synergistic antitumor effect of C + o was dependent on CD8^+^ T cells.

**Conclusion:**

The treatment strategy of CAR-Ms combined with oAd-CD47 provides a promising, novel and effective treatment method for individualized targeted therapy of solid tumors.

**Supplementary Information:**

The online version contains supplementary material available at 10.1186/s40164-025-00696-7.

## Graphical Abstract

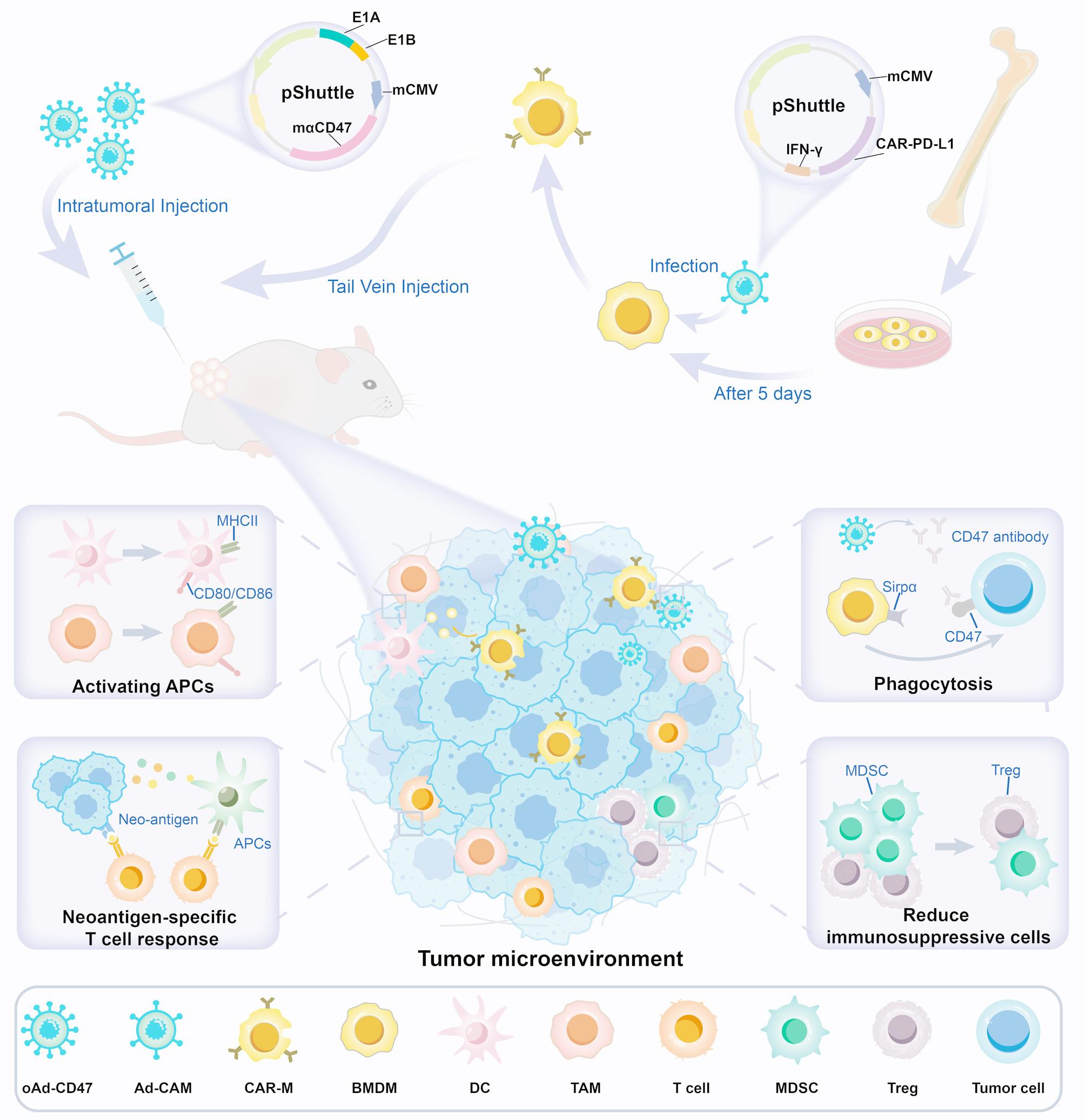

## Background

Chimeric antigen receptor (CAR)-T cell therapy has gained considerable success in the treatment of hematopoietic malignancies [1]. CAR is an engineered synthetic receptor that provides T cells with the ability to recognize and destroy cells expressing specific target antigens [2]. While CAR-T cell therapy has notable curative effects in treatment of hematopoietic malignancies [3, 4], its therapeutic value in the treatment of solid tumors remains uncertain and is constrained by several challenges [5]. First, solid tumors have limited tumor-specific antigen (TSA) targets, thereby complicating the development of CAR-T therapies with high specificity and minimal off-target effects. Secondly, the presence of dense and complex extracellular matrix (ECM) and abnormal blood vessels within solid tumors limit the migration and infiltration of CAR-T cells into tumor sites [6]. Furthermore, presence of immunosuppressive cells and molecules within the tumor microenvironment hinder the functionality of CAR-T cells [7]. These limitations significantly impede effective application of CAR-T therapy for treating solid tumors, thereby necessitating further in-depth research and innovative strategies to address these challenges.

Macrophages are important components within the immune system, contributing to the host defense through mechanisms such as inflammation [8]. These cells can modify their phenotype in response to environmental changes and are typically divided into two functional categories: classically activated macrophages (M1) and alternatively activated macrophages (M2) [9]. Macrophages infiltrate tumor tissues where immunosuppressive tumor microenvironment is characterized by low oxygen, nutrients deficiency, and high lactate levels, resulting in the formation of tumor-associated macrophages (TAMs) [10]. Generally, TAMs are regarded as unique phenotype of M2-like macrophages [11]. Substantial evidence indicate that TAMs mediate immune suppression: by secreting immunosuppressive cytokines; by recruiting immunosuppressive cells including regulatory T cells (Tregs) and myeloid suppressive cells (MDSCs) to the tumor microenvironment; and by enhancing tumor immune evasion [12]. Currently, several therapeutic strategies targeting macrophages have been developed, including those focused on reducing or eliminating TAMs, repolarizing TAMs into M1-like macrophages, blocking inhibitory receptors on TAMs, and inhibiting the self-recognition signal on TAMs [13, 14]. Among these strategies, CAR-Ms have emerged as a promising tool in cancer immunotherapy as it addresses the challenges associated with CAR-T and CAR-NK cell therapies [15]. Currently, CAR-Ms are undergoing clinical evaluation [16, 17].

The Programmed Cell Death 1 Receptor (PD-1)/Programmed Cell Death Ligand 1 (PD-L1) pathway is a recognized tumor immune inhibition pathway [18]. PD-L1 is highly expressed in various malignant tumors. Currently, antibodies targeting the PD-1/PD-L1 pathway are used in treatment of various cancers, such as lung, liver, and colorectal cancers, along with melanoma [19, 20]. CD47 is a significant transmembrane glycoprotein commonly expressed on cancer cells. It interacts with the inhibitory receptor signaling regulator alpha (SIRPα) to transmit a self-recognition signal that inhibits phagocytosis, thereby leading to macrophage dysfunction [21]. In response, several therapeutic antibodies and proteins targeting CD47-SIRPα have been developed to enhance the phagocytic activity of macrophages [22, 23]. In our previous studies, we engineered an oncolytic adenovirus encoding CD47 antibodies (oAd-CD47), designed to enhance macrophage-mediated phagocytosis by blocking CD47 expression on tumor cells [24]. The CD47-SIRPα pathway, which acts as an inhibitory pathway to both recognition and phagocytic functions of macrophages, has been targeted in the development of novel macrophage-based therapies [16].

Interferon-gamma (IFN-γ) signal transduction is vital for pro-inflammatory activation of macrophages [25]. In a study by Shields et al., an engineered particle integrated with IFN-γ was used to induce and maintain polarization of macrophages to adopt an antitumor phenotype, which highlights the significance of initiating and maintaining the macrophage therapeutic phenotype for effective cancer treatment. These findings informed our approach to induce M1 polarization in CAR-Ms [26]. In this study, we engineered CAR-Ms targeting the PD-L1 antigen using a non-replicative adenoviral vector and then encoding the PD-L1-CAR structure with IFN-γ gene. We demonstrated the phagocytic activity of CAR-Ms on PD-L1^+^ tumor cells in vitro. Phenotypically, they were similar to M1 macrophages characterized by overexpression of MHC II and CD68. Furthermore, we investigated CAR-Ms anti-tumor efficacy in two homologous transplant mouse tumor models and preliminarily explored the underlying immunological mechanisms. Additionally, we explored Oncolytic adenovirus expressing the CD47 antibody (oAd-CD47) potential to enhance CAR-Ms antitumor efficacy in preclinical tumor model. The combined therapeutic strategy demonstrated robust antitumor efficacy, not only in two distinct mouse subcutaneous tumor models but also in an ID8 ascites-bearing metastatic ovarian cancer model. This effect was primarily mediated by CD8⁺ T cells, as confirmed in the CT26 subcutaneous tumor model. Additionally, we discovered that this strategy enhanced phagocytosis of macrophages within the tumor microenvironment and triggered neoantigen specific T cell responses. This study provides preclinical evidence supporting the clinical translation of the CAR-Ms and oAd-CD47 combination therapy.

## Methods

### Cell lines

The CT26, B16-F10 and ID8 cell lines were acquired from the American Type Culture Collection (ATCC). They were maintained in RPMI 1640/DMEM (HyClone) media supplemented with 10% fetal bovine serum (FBS) (HyClone) at 37 °C. Subsequently, ID8-luc cell lines were generated using viral transduction followed by selection with puromycin (1 µg/mL). Similarly, ID8 cells expressing green fluorescent protein and ovalbumin gene (ID8-EGFP-OVA) were generated using the same method. All cell lines were confirmed to be mycoplasma-free.

### Preparation of bone marrow-derived macrophages (BMDMs)

Flushing of femurs and tibias of mice using DMEM was conducted to extract bone marrow cells. The resulting cell suspension was filtered using a 70 μm filter, then washed twice with PBS. The obtained cells were then cultured in complete medium containing 30% L929 cell supernatant. BMDMs were collected for experimentation on day 5.

### Plasmid, virus and CAR-Ms

The replication-deficient adenoviral shuttle vector (pDC316-mPD-L1nb-CAR) was synthesized by Genewiz. HEK293A cells were co-transfected with shuttle vector containing CAR-mPD-L1-IFN-γ structure and a backbone plasmid for packaging the non-replicative adenovirus (Ad-CAM). The empty control vector (Ad-ON) retained the same backbone but lacked the CAR-mPD-L1-IFN-γ insert. Viral particles were amplified in HEK293 cells and purified by CsCl gradient centrifugation followed by dialysis using dialysis buffer. The adenoviral titer was determined using 50% tissue culture infection volume (TCID50) method.

The oncolytic adenovirus (oAd-CD47) was constructed as previously described [24]. The E1A-IRES-E1B gene was inserted into pDC316 under the control of the human telomerase reverse transcriptase promoter to construct an oncolytic adenovirus shuttle vector (pDC316-hTERT-E1A-IRES-E1B). The transgene mCD47nb-Fc was cloned into the aforementioned oncolytic adenovirus shuttle vector under the control of the mCMV promoter (pDC316-hTERT-E1A-IRES-E1B-mCD47nb-Fc). The plasmid pDC316-hTERT-E1A-IRES-E1B-mCD47nb-Fc was co-transfected with the adenoviral backbone plasmid into 293 A cells to generate the recombinant virus. Viral amplification was subsequently carried out in HEK293 cells, and viral particles were purified by cesium chloride (CsCl) gradient centrifugation followed by dialysis with a specialized buffer. The final adenoviral titer was quantified using the 50% tissue culture infectious dose (TCID₅₀) method.

Ad-ON and Ad-CAM were administered to infect BMDMs at a multiplicity of infection (MOI) of 50 to generate control macrophages (Con-Ms) and CAR-Ms, respectively. The MOI is defined as the ratio of plaque-forming units (PFU) to target cells.

### In vitro phagocytosis assay

The phagocytosis assay was performed in ultra-low attachment 24-well plates (Corning, 3473) to minimize tumor cell adherence. Briefly, DiD (a cell membrane fluorescent dye provided by Abkine)-labeled tumor cells (5 × 10⁴/well) were co-cultured with CAR-M cells (effector-to-target ratio = 2:1) for 5 h in RPMI-1640 supplemented with 10% FBS at 37 °C under 5% CO₂. To further prevent adherence, cells were gently resuspended every 30 min during the 4-h incubation. Phagocytosis was quantified by flow cytometry (FCM) (DiD⁺F4/80⁺ cells) and normalized to control wells (CAR-M alone).

Similarly, (CFDA-SE) (5 (6)-carboxydiacetate fluorescein succinimidyl ester) (CFSE)-labeled BMDM cells were incubated with DiD-labeled CAR-M (effector-to-target ratio = 1:1) for 5 h in RPMI-1640 supplemented with 10% FBS at 37 °C under 5% CO₂. Phagocytosis was quantified by FCM (the percent of DiD⁺ cells under CSFE gate) and normalized to control wells.

### Co-incubation experiments with M2-type macrophages

On the fifth day of BMDM culture, IL-4 (20 ng/mL) was added to the medium and incubated for 48 h to induce M2 polarization. After induction, M2 macrophages were harvested and labeled with CFSE (5 µM) for 20 min at 37 °C. Following the staining process, the M2 macrophages were co-cultured with Con-M or CAR-M for 48 h. Flow cytometry was performed using anti-iNOS and anti-Arg1 antibodies, and the proportion of Arg1⁺/iNOS⁻ cells was measured to quantify the M2 macrophage population.

### CAR-Ms migration assays

Migration assays for CAR-Ms were performed using 24-well plates equipped with 5.0 μm polycarbonate membrane inserts (LABSELECT, 14331-D). Briefly, 5 × 10^4^ CAR-Ms were placed in the upper chambers containing 200 µL of RPMI 1640 supplemented with 2% FBS. The CAR-Ms were then co-cultured with 1 × 10^5^ tumor cells (infected with oAd-CD47 or left uninfected). After 3 days, the cells remaining in the upper chamber were removed using cotton swabs, and the membrane of the insert was stained with crystal violet and imaged. The number of CAR-Ms that migrated through the membrane was quantified by counting the stained cells.

### RNA sequencing

Extraction of RNA from the Con-M and CAR-M samples was conducted and a Bioanalyzer used to assess the integrity of extracted RNA. Subsequently, RNA sequencing was done by the sequencing and microarray facility, located at the Institute of Life Sciences, using an Illumina sequencer. HISAT2 RNA-seq alignment software was used to align raw sequencing reads to the mm10 reference genome (build mm10). The Feature Counts tool in Subread software was used to quantify gene expression levels. Differential expression analysis of the count data was performed using R software package DESeq2, applying a threshold of |log2(Fold Change)| ≥1 and padj ≤ 0.05 to identify differentially expressed genes. ClusterProfiler software was used to perform functional enrichment analysis for gene ontology (GO), and KEGG pathway enrichment analysis of the differentially expressed gene sets.

### Real-time PCR

RNA was extracted using TaKaRaMiniBEST Universal RNA Extraction Kit (TaKaRa) and then reverse transcribed using PrimeScript™ RT reagent Kit with gDNA Eraser (TaKaRa). SYBR Green III (Vazyme) was used to perform RT-qPCR. The primer sequences were listed as follows:

Spp1: AGCAAGAAACTCTTCCAAGCAA, GTGAGATTCGTCAGATTCATCCG Mrc1: CTCTGTTCAGCTATTGGACGC, CGGAATTTCTGGGATTCAGCTTC Nos2: GTTCTCAGCCCAACAATACAAGA, GTGGACGGGTCGATGTCAC Cxcl10: CCAAGTGCTGCCGTCATTTTC, GGCTCGCAGGGATGATTTCAA.

Cd86: TGTTTCCGTGGAGACGCAAG, TTGAGCCTTTGTAAATGGGCA.

Cd74: AGTGCGACGAGAACGGTAAC, CGTTGGGGAACACACACCA.

Mertk: CAGGGCCTTTACCAGGGAGA, TGTGTGCTGGATGTGATCTTC.

Fcgr1: AGGTTCCTCAATGCCAAGTGA, GCGACCTCCGAATCTGAAGA.

### Animal studies

Six-week-old C57BL/6J or Balb/c female mice were obtained from Beijing Huafukang Bioscience (Beijing, China). All animal experiments were performed following the guidelines by the Animal Care and Use Committee of West China Hospital, Sichuan University, China.

In the B16 and CT26 subcutaneous tumor models, B16 or CT26 tumor cells (1 × 10^6^ cells suspended in 100 µL DMEM) were injected into the right flank of the mice. Once the tumor volume reached about 100 mm^3^, the mice were randomized. CAR-Ms or Con-Ms (2 × 10^6^cells suspended in 200 µL DMEM) were injected into the tail vein three times, with a three-day interval between each injection. The mice were intratumorally injected with the oAd-CD47 virus at a dose of 2 × 10^8^ PFU. The length and width of the tumor were measured every two days using vernier caliper and the tumor volume calculated using the formula: tumor volume = length×(width)^2^ × 0.52.

For the ID8 peritoneal metastasis model, ID8-Luc tumor cells (8 × 10^6^ cells suspended in 500 µL DMEM) were injected intraperitoneally into mice. After confirming tumor formation, mice were randomly assigned and injected intraperitoneally with CAR-Ms (4 × 10^6^ cells suspended in 500 µL DMEM) and the oAd-CD47 virus with a dose of 0.8 × 10^8^ PFU. Tumor progression was monitored in real time using an IVS50 bioluminescence imaging system (PerkinElmer) and analyzed with Live Image 2.6 software (PerkinElmer).

### Biodistribution evaluation of CAR-Ms

The biodistribution evaluation of CAR-Ms was conducted on CT26 subcutaneous tumor model mice. The macrophages used for injection were labeled with a cell membrane near-infrared fluorescent probe (DIR). Three days post the final injection, the tumor tissues were excised and prepared for ex vivo imaging.

### In vivo phagocytosis assay

Mice bearing CT26-mCherry subcutaneous tumor were treated as described in Sect. 2.9 above. Three days after the final injection, tumor tissues were excised and prepared into single-cell suspensions. Anti-CD45, anti-CD11b and anti-F4/80 was used for staining the cells and analysis performed using FCM. The percentage of mCherry^+^ F4/80^+^ cells was calculated as percentage of phagocytic activity. Similarly, ascites was collected from mice implanted with ID8-EGFP-OVA cell line as described in Sect. 2.8 and then single-cell suspensions were prepared after lysing erythrocytes. The anti-F4/80 was used to stain the cells and analysis performed using FCM. The percentage of EGFP^+^ F4/80^+^ cells was calculated to indicate phagocytosis. To investigate the role of CAR-M in the TME, the dosing regimen outlined in Sect. 2.8 was followed. DiR, a fat-soluble near-infrared fluorescent dye, was used to label CAR-M for therapy. Following the collection of a single-cell suspension, cells were stained with anti-F4/80 and anti-CD206 antibodies. The proportion of CD206⁺ DiR⁺ cells was used to identify M2-type macrophages, while the proportion of EGFP⁺ cells within the F4/80⁺ gate, further filtered by the DiR⁺ gate, was used to measure phagocytosis.

### Flow cytometry analysis

The following staining protocol was used to assess CAR-PD-L1 expression on CAR-Ms: after 10-min Fc-blocking using BD Biosciences, cells were stained with Recombinant Mouse PD-L1-his (Novoprotein) and FITC anti-mouse F4/80 antibody, followed by His-tag Mouse mAb (ZEN BIO) and Rabbit Anti-mouse IgM/PE-Cy7. The following panel was used to assess the phenotypic characteristics of NC-M and CAR-Ms: anti-F4/80 FITC, anti-Arg1 PE-Cy7), and anti-iNOS APC. Additionally, the following panel was used to assess the functional analysis of NC-M and CAR-Ms: anti-F4/80 FITC, anti-MHCII PE, and anti-CD86 APC. Anti-CD47 PE and Anti-PD-L1 APC were used for CT26 and B16 tumor cells surface staining.

For analysis of the tumor and spleen microenvironment, tumor and spleen tissues were collected from mice with CT26 subcutaneous tumor model three days after the final treatment. To prepare single-cell suspensions from tumor tissue, approximately 50 mg of the resected tumor was cut into 1–2 mm³ fragments using sterile surgical scissors. The tissue was then digested in RPMI-1640 medium containing collagenase IV (1 mg/mL; Sigma, C5138) and DNase I (20 µg/mL; Roche, 10104159001) at 37 °C for 45 min with gentle agitation (150 rpm). Following enzymatic digestion, the suspension was filtered through a 70 μm cell strainer (Corning, 352350) to remove residual undigested material. Similarly, half of the spleen tissues harvested were ground with a syringe plunger and filtered through a 70 μm strainer followed by red blood cells lyses to produce a single cell suspension. The cells were then blocked with CD16/CD32 antibody and dead cells were excluded using Zombie Violet. Cell surface staining was performed at 4 °C in PBS in the dark for 25 min. For intracellular staining, the cells were fixed and permeabilized with BD Cytofix/Cytoperm Plus Fixation/Permeabilization Kit (BD Biosciences). After washing twice, the cells were resuspended in PBS for antibody staining. Flow cytometry analysis was performed using a BD Biosciences LSRFortessa, and the data analyzed using flowjo software (Tree Star Inc.). The following antibodies were used in cell staining:

Panel 1 was used for T cells staining in tumor: CD3-Texas Red, CD4-BUV395, CD8-PE-Cy7, PD-1-Percp, Tim3-PE, CD25-FITC, and Foxp3-APC.

Panel 2 was used for TAMs/dendritic cells (DCs)/MDSCs: CD45-APC-cy7, CD11b-APC-cy5, F4/80-BUV730, CD206-PE-cy7, MHCII-BV711, GR1-BV786, CD86-APC, CD11c-BV650, and CD80-FITC.

Panel 3 was used for T cells staining in spleen: CD3-Texas Red, CD4-FITC, CD8-BV510, TNF-α-APC, and IFN-γ-PE.

All antibodies for flow cytometric analysis were obtained from Biolegend. Samples for assessing IFN-γ and tumor necrosis factor-alpha (TNFα) were stimulated with phorbol 12-myristate 13-acetic acid (PMA) and ionomycin for 2 h before fixation and staining.

### OVA specific-T cell response assay

ID8-EGFP-OVA tumor cells (8 × 10^6^ cells suspended in 500 µL DMEM) were injected intraperitoneally into mice. Seven days after the final treatment, spleens were aseptically excised and lymphocyte suspensions were harvested. Lymphocytes were plated at a density of 1 × 10^5^ cells per well in anti-IFN-γ precoated plate and incubated with OVA peptide (final concentration: 10 µg/ml) for 24 h at 37 °C. IFN-γ production was assessed using Mouse IFN-γ ELISpot kit and visualized using ImmunoSpot S6 Universal. Quantification of IFN-γ was performed using ImmunoSpot V.7.0.15.0 Professional Analyzer DC software.

### Neoantigen-specific T cell response assays

Spleens were prepared as discussed in 2.12 above and seven days after treatment, spleen were aseptically excised and prepared and lymphocyte suspensions harvested. Lymphocytes were plated at a density of 1 × 10^5^ per well in 96-well plates and incubated for three days with OVA peptide (final concentration: 10 µg/ml), 6 neoantigen peptides, and mixed peptide pools (final concentration of each antigen peptide: 2 µg/ml) in the presence of complete medium supplemented with 100 µg/ml double antibodies, 50 µM 2-mercaptoethanol, and 300 IU/ml recombinant mouse IL-2, and 100 µL supernatant was collected from each well and IFN-γ expression levels assessed using Mouse IFN-γ Precoated ELISA kit (YOTA. The remaining cells were used for Edu-488 cell proliferation assay kit (Beyoclick) to assess T cell proliferation after neoantigen stimulation. CT26 neoantigen peptides were synthesized using Fmoc-based solid-phase synthesis (GL Biochem, Shanghai), incorporating N-terminal acetylation and C-terminal amidation. The synthesized peptides were purified to > 95% purity via reversed-phase HPLC), and their identities were verified by MALDI-TOF mass spectrometry. The sequence of the neoantigen peptide is presented as follows [27]: 1: Aldh18a1: HSGQNHLKEMAISVLEARACAAAGQ; 2: E2f8: ILPQAPSGPSYATYLQPAQAQMLTP; 3: Ndc1: HSFIHAAMGMAVTWCAAIMTKGQYS; 4: Slc20a1: KPLRRNNSYTSYIMAICGMPLDSFR; 5: Mtch1: SWIHCWKYLSVQSSQLFRGSSLLFRR; 6: Dhx35: VIQTSKYYMRDVIAIESAWLLELAP.

### In vivo T cell depletion experiments

Anti-mouse CD4-Invivo (Selleck) and anti-mouse CD8-Invivo (Selleck) were injected intraperitoneally at a dose of 200 µg per mouse every three days, for a total of three days, starting at two days before treatment initiation. Three days after the first injection, peripheral blood monocyte suspension was obtained and stained with anti-mouse CD3, CD4, and CD8 antibody to assess the blocking efficacy. Tumor assessment procedures were conducted as described in Sect. 2.13.

### Statistical analysis

Statistical analyses were performed using Graphpad prism software version 8.0 and IBM SPSS software version 27. A two tailed Student’s t-test was used for comparison between the two groups, whereas one-way analysis of variance (ANOVA) was applied for multiple group comparisons. All experiments were independently repeated [n] times with consistent results, where [n] represents the biological replicates. The figure presents representative data from at least three independent experiments (*n* ≥ 3), with the data expressed as the mean ± the standard error of the mean (SEM). Significance levels were denoted as, *p < 0.05, **p < 0.01, ***p < 0.001, and N.S for not significant.

## Result

### Construction of CAR-Ms and characterization their phagocytic capacity and phenotype in vitro

**Fig. 1:**
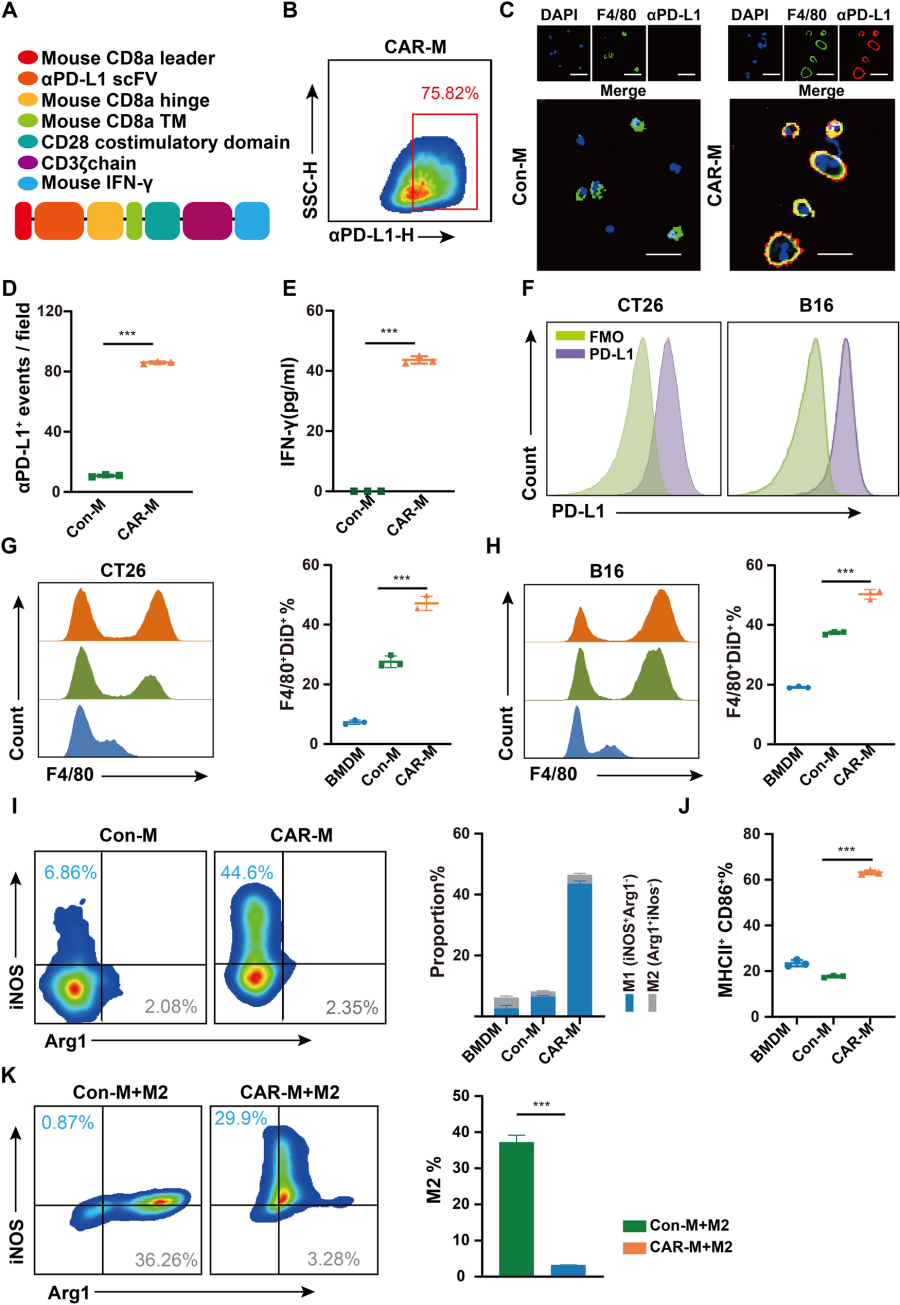
Generation of CAR-Ms and evaluation of their phagocytosis and phenotypic. A. Constructs utilized in adenovirus vector to express CAR-PD-L1 and IFN-γ in BMDMs. B. Representative flow cytometry (FCM) plot showing the CAR-PD-L1 expression on BMDMs infected with Ad-CAM (CAR-Ms). C. Representative images of CAR-PD-L1 expression on Con-Ms and CAR-Ms in each field of view taken using a microscope at 20× from Con-Ms and CAR-Ms. Scale bars are 50 μm. D. The numbers of CAR-PD-L1^+^ macrophages in each field. The cell counts were obtained from at least three fields. E. The expression of IFN-γ in CAR-Ms. F. Representative FCM plot showing the expression of PD-L1 on CT26 and B16 tumor cells. G. FCM-based phagocytosis of CT26 tumor cell by BMDMs, Con-Ms and CAR-Ms. Histogram of F4/80 expression under DiD⁺ gates (Left). A statistical plot presenting the percentage of DiD⁺F4/80⁺ cells (Right). H. FCM-based phagocytosis of B16 tumor cell by BMDMs, Con-Ms and CAR-Ms. Histogram of F4/80 expression under DiD⁺ gates (Left). A statistical plot presenting the percentage of DiD⁺F4/80⁺ cells (Right). I. Representative FCM plot indicating the iNOS and Arg1 expression on Con-Ms and CAR-Ms. The proportion of M1 (iNOS^+^ Arg1^-^) and M2 (iNOS^-^ Arg1^+^) macrophages in BMDMs, Con-Ms and CAR-Ms. J. The percentage of MHCII+ CD86+ macrophages in BMDMs, Con-Ms and CAR-Ms. K. IL-4-induced M2 macrophages were co-cultured with Con-Ms and CAR-Ms for 48h. CFSE was used to distinguish il-4 induced M2 macrophages from other cells. Representative flow plots of iNOS and Arg1 expression in Con-M and CAR-M are shown on the left. On the right is its statistical histogram. In all panels, statistical significance was calculated using unpaired t-test and one-way ANOVA with multiple comparisons, and data are presented as the mean ± SEM. from three independent experiments. *P < 0.05, **P < 0.01, ***P < 0.001.

We engineered an anti-PD-L1 CAR and IFN-γ structures into an adenoviral vector (Fig. 1A) and infected BMDMs to generate CAR-Ms. BMDMs infected with empty adenovirus vector served as control macrophages (Con-Ms). We demonstrated that CAR-Ms expressed CAR-PD-L1 with high efficacy (Fig. 1B-D) and secreted IFN-γ (Fig. 1E). The results of FCM analysis revealed that adenovirus vector infection did not cause cell death in either Con-M or CAR-M (Fig. S1A, B). FCM analysis showed that murine colon cancer cell line CT26 and melanoma cell line B16 highly expressed PD-L1 (Fig. 1F). These two cell lines were co-cultured with BMDMs, Con-Ms, and CAR-Ms, to evaluate their phagocytic activity. FCM analysis demonstrated that the phagocytic capacity of CAR-Ms was significantly enhanced compared to those of BMDMs and Con-Ms (Fig. 1G and H). We found that CAR-Ms exhibited a pro-inflammatory M1-like phenotype (Arg1^-^iNOS^+^) (Fig. 1I) and highly expressed MHCII and CD86 (Fig. 1J). We co-incubated IL4-induced M2-like macrophages with Con-Ms and CAR-Ms to assess the plasticity of their phenotypes, with the results indicating that Con-Ms were more susceptible to M2-like polarization compared to CAR-Ms (Fig. 1K).

### Pro-inflammatory characteristics of CAR-Ms at the genetic level

**Fig. 2:**
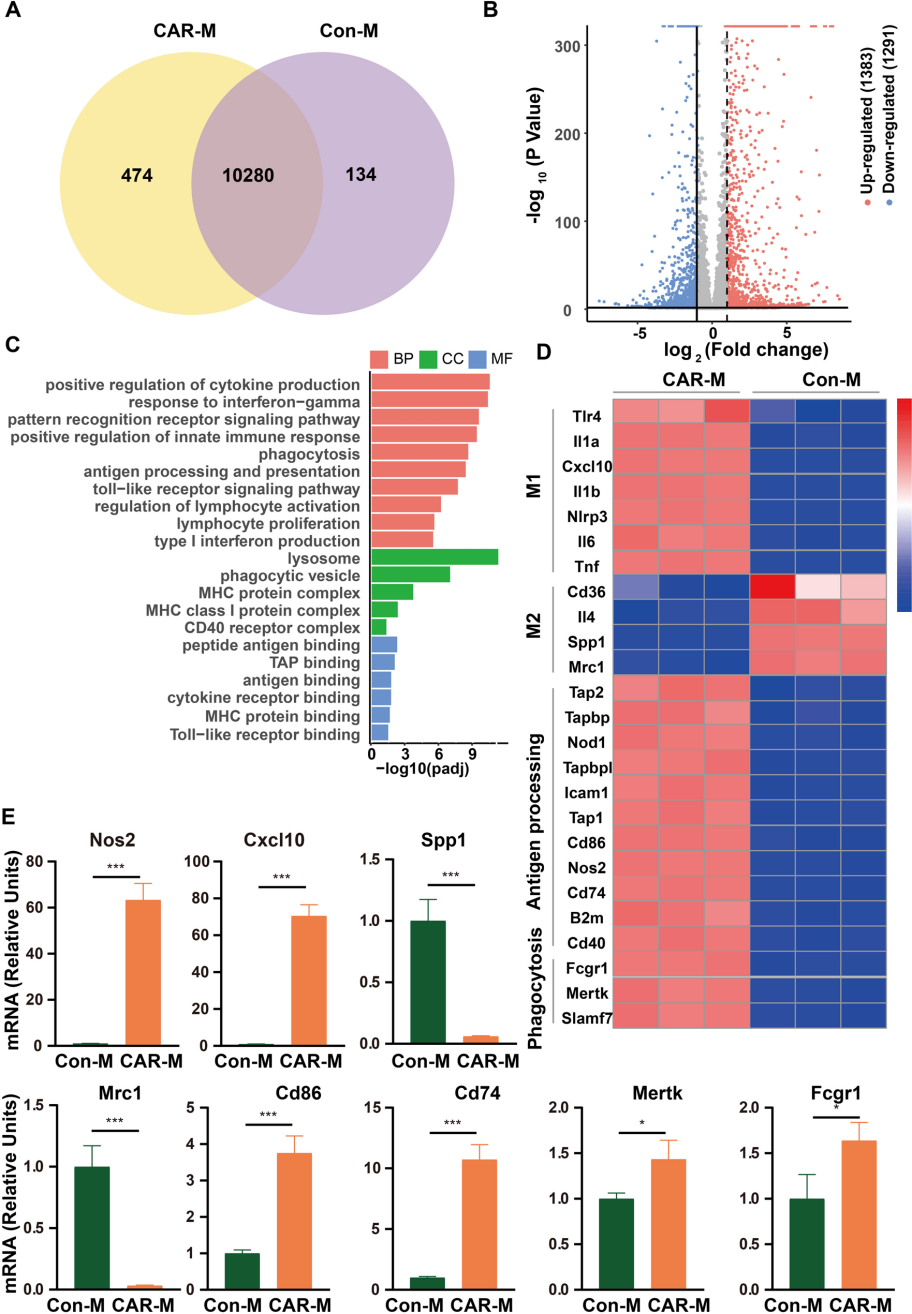
CAR-Ms exhibit a pro-inflammatory (M1) macrophage phenotype. **(A)** Clustering by principal component analysis of gene expression from Con-Ms and CAR-Ms. *n* = 3. **(B)** Volcano plot of differentially expressed genes in Con-Ms versus CAR-Ms. Red and blue indicate Padj < 0.05 and log2 fold change > 1 or < − 1. *n* = 3. **(C)** Gene Ontology (GO) analysis of differentially regulated genes in Con-Ms and CAR-Ms. *n* = 3. Biological Process (BP), Cellular Component (CC) and Molecular Function (MF) are three term categories of GO, used for systematic annotation and enrichment analysis of gene functions. **(D)** Heatmap showing the differentially expressed M1 marker, M2 marker co-stimulatory ligands, antigen processing genes, and phagocytosis gene between Con-Ms and CAR-Ms via RNA-seq. **E.** Confirmation of select RNA-seq results in (2D) via RT-qPCR. Data are presented as the mean ± SEM. from three independent experiments. Statistical analysis was calculated using unpaired t-test. In all panels, *P < 0.05, **P < 0.01, ***P < 0.001

We conducted transcriptome sequencing (RNA sequencing) on Con-Ms and CAR-Ms to highlight their genotype profiles. The differential gene expressions (DEGs) were filtered and identified. As shown in Figs. 2A, 10 and 280 DEGs were identified between CAR-Ms and Con-Ms. CAR-Ms upregulated 1,383 genes and downregulated 1291 genes compared to Con-Ms (Fig. 2B). The GO analysis of the upregulated genes revealed significant enrichment in pathways associated with pro-inflammatory responses, phagocytosis, and antigen processing in CAR-Ms (Fig. 2C). Consistent with the in vitro phenotype data, the gene expression heatmap revealed that M1-associated markers were up-regulated whereas M2-associated markers were down- regulated (Fig. 2D). Furthermore, costimulatory ligands, antigen processing genes, MHCII antigens, and phagocytosis-associated markers were significantly upregulated (Fig. 2D). Validation of the representative DEGs using RT-qPCR confirmed the RNA-seq findings (Fig. 1E).

### CAR-Ms inhibit tumor progression and prolong survival of tumor-bearing mice

**Fig. 3:**
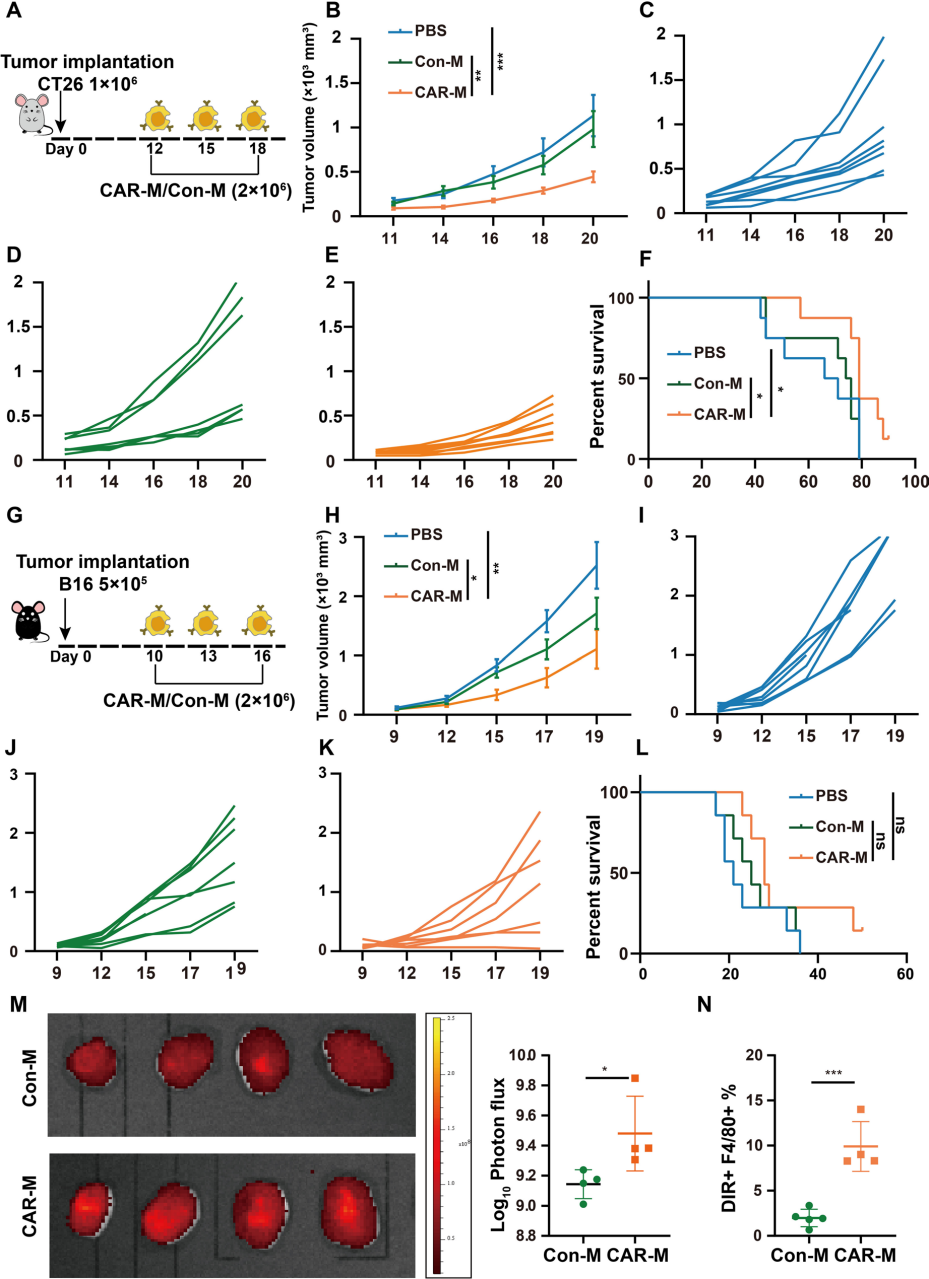
Evaluation of anti-tumor activity CAR-Ms in CT26 and B16 subcutaneous tumor model. **(A)** Experimental timeline. Tumor-bearing mice received three intravenous injections of PBS, Con-Ms, or CAR-Ms on days 12, 15, and 18 after CT26 tumor cell inoculation. **(B)** The mean volume of tumor growth. Tumor volume of PBS group (**C**), Con-Ms group (**D**) and CAR-Ms group (**E**) measured every two days. Statistical significance of tumor volume was calculated with one-way ANOVA using multiple comparisons, and data represent *n* = 6–8. **F.** Kaplan-Meier survival curve of mice bearing CT26 tumor. Statistical significance of survival was calculated using the log-rank Mantel-Cox test with df = 2. **G.** Experimental timeline. On days 10, 13 and 16 after subcutaneous injection of B16 tumor cells, PBS, Con-Ms and CAR-Ms were injected into the tail vein of tumor-bearing mice. **H.** The mean volume of tumor growth. Tumor volume of PBS group (**I**), Con-Ms group (**J**) and CAR-Ms group (**K**) measured every two days. **L.** Kaplan-Meier survival curve of mice bearing B16 tumor. **M.** Accumulation of DIR - labeled Con-Ms and CAR-Ms in tumor tissues of CT26 tumor-bearing mice after three intravenous injections. Mice were killed and tumors were explanted for ex vivo imaging 5 d after the last injection of 2 × 10^6^ Con-Ms or CAR-M. And statistical histogram of total flux (p/s) in different groups. **N.** FCM-based accumulation of DIR-labeled Con-Ms and CAR-Ms in CT26 tumor tissues. Statistical analysis was performed with unpaired t-test and data are presented as the mean ± SEM. of (*n* = 4 mice). In all panels, *P < 0.05, **P < 0.01, ***P < 0.001

Two mice tumor models with different immunogenicity were used to investigate the anti-tumor efficacy of CAR-Ms in vivo. Mice bearing highly immunogenic CT26 colorectal cancer received three intravenous injections of PBS, Con-Ms, and CAR-Ms (Fig. 3A). Tumor burden was significantly reduced in CAR-M-treated mice (Fig. 3B-E). Although tumor progression occurred in all groups, CAR-M injection extended overall survival (Fig. 3F). In a second model, B16-F10 cells were injected subcutaneously into C57BL/6J mice (Fig. 3G). The CAR-M showed a weak anti-tumor response against B16-F10 melanoma with poor immunogenicity. CAR-Ms significantly inhibited tumor growth compared with the Con-Ms (Fig. 3H-K), but without significant improvement in overall survival (Fig. 3L).

Next, we evaluated the bio-distribution of CAR-Ms after systemic administration. DIR was used to label CAR-Ms and Con-Ms to track their distribution in tumor-bearing mice after treatment. To evaluate the distribution of CAR-Ms, relative fluorescence signals in tumors, liver, spleen, and lung were evaluated ex vivo five days after administration of DIR–labeled CAR-Ms (Fig. S2A, B). The results indicated that CAR-Ms were successfully transported to the tumor site (Fig. 3M, N), with the liver being the main site of macrophage accumulation after tail vein administration. Meanwhile, pathology of organ tissue did not indicate organ damage (Fig. S2C). Additionally, intravenous injection CAR-Ms carrying IFN- γ did not induce systemic toxicity as revealed by normal serum levels of liver enzymes (aspartate aminotransferase and alanine aminotransferase) and kidney function markers (creatinine and blood urea nitrogen) (Fig. S2D).

### CAR macrophage targeting PD-L1 remodels tumor microenvironment

**Fig. 4:**
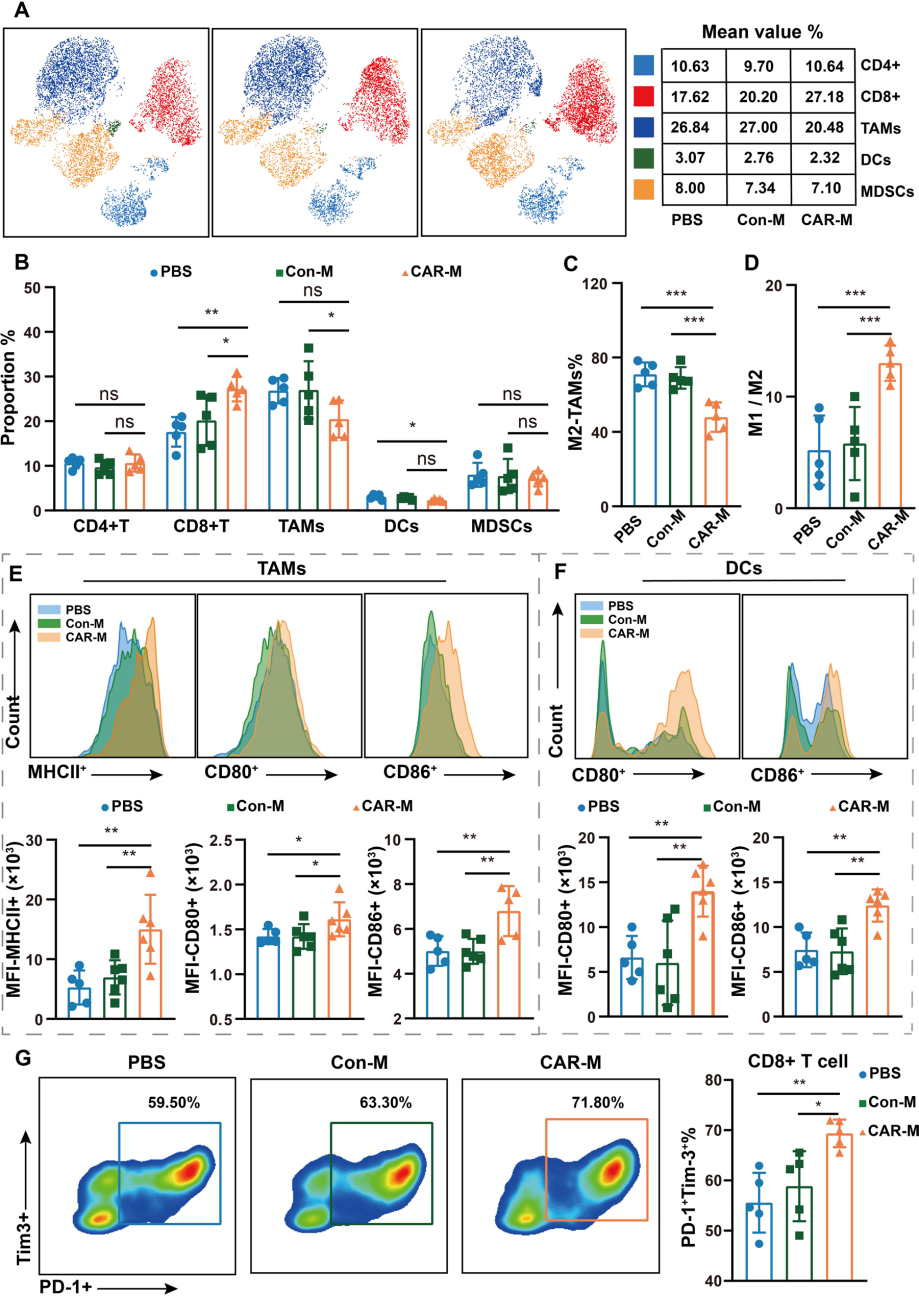
TME analysis of CT26 tumor-bearing mice after treated with PBS, Con-Ms and CAR-Ms. **(A)** T-SNE analysis of CD4^+^ T cell, CD8^+^T cell, TAMs, DCs and MDSCs in TME. **(B)** The percentage of CD4^+^ T cell, CD8^+^T cell, TAMs, DCs and MDSCs in the TME. **(C)** The percentage of M2 macrophages (marked by CD206^+^) in the TME. **(D)** The ratio of M1 and M2 in the TME. **(E)** Representative FCM plot for MHCII^+^, CD80^+^, CD86^+^ in TAMs. The mean fluorescence intensity (MFI) of MHCII^+^, CD80^+^, CD86^+^ cells in TAMs. **(F)** Representative FCM plot of CD80^+^ and CD86^+^ in DCs. And MFI of CD80^+^ and CD86^+^ cells in DCs. **(G)** Representative FCM plot of PD-1^+^ Tim3^+^ in CD8^+^ T cell. The percentage of PD-1^+^ Tim3^+^ cells in CD8^+^ T cell. In all panels, statistical significance was calculated using one-way ANOVA with multiple comparisons, and data are presented as the mean ± SEM. of *n* = 5 mice. *P < 0.05, **P < 0.01, ***P < 0.001

To investigate the immune mechanism of the anti-tumor efficacy in CAR-Ms, we conducted FCM analysis on immune cells in tumors. We analyzed the composition of immune cells in TME after CAR-Ms treatment (Fig. 4A). The results showed that CAR-Ms significantly increased the percentage of CD8 + T cells but did not have any effect on CD4 + T cells (Fig. 4B). We further investigated the changes in the antigen-presenting cell (APC) population after CAR-Ms treatment except for the T cell population. Accumulation of TAMs in the CAR-Ms group significantly decreased compared to the Con-M group, while no significant difference was observed in the group for dendritic cells (DCs). Moreover, compared to the Con-M and PBS groups, the CAR-M group exhibited a marked reduction in the number of M2-like macrophages (Fig. 4C), along with a significant elevation in the M1/M2 ratio (Fig. 4D). Consistently, a heat map analysis of CD206 expression revealed a noticeable downregulation of CD206 levels (Fig. S3A). The expression of antigen-presenting biomarker (MHCII^+^, CD86^+^, CD80^+^) on macrophages and DCs was significantly enhanced in tumors treated with CAR-Ms, compared to the Con-Ms group tumor (Fig. 4E, F, Fig. S3B, C), indicating enhanced maturation and antigen-presenting function.

Immmunosuppressive cell types within the TME, including MDSCs and Treg, can inhibit the function of other immune cells [28]. To verify whether CAR-Ms can further polarize the surrounding microenvironment, we investigated the expression levels of MDSCs and Treg in TME. The results showed that CAR-Ms did not significantly reduce the number of MDSCs and Treg cells (Fig. 4B and Fig. S3F). Notably, the increased accumulation of PD-1^+^Tim-3^+^CD8^+^T cells within CAR-Ms treated tumors implies that CD8^+^T cells are in a state of functional exhaustion (Fig. 4G and Fig. S3F, G), whereas PD-1^+^Tim-3^+^CD4^+^T cells remained unchanged (Fig. S3E). These findings suggest that the enhanced anti-tumor efficacy of CAR-Ms is primarily driven by their ability to repolarize TAMs and activate DCs.

### oAd-CD47 enhances phagocytosis of CAR-Ms in vitro and promotes migration of CAR-Ms

CD47 is often upregulated in tumor cells, where it interacts with SIRPα on surfaces of macrophages, to release the self-recognition signal [29], and thereby evading CAR-Ms phagocytic activity. CD47 blocking disrupts the CD47-SIRPα axis, which mediates immunosuppressive signaling. Competitive binding of anti-CD47 antibodies to CD47 on tumor cells blocks the interaction between CD47 and SIRPα on CAR-Ms, thereby reversing macrophage immune suppression and significantly enhancing the phagocytic activity of CAR-Ms. Additionally, the introduction of oncolytic viruses enhances immune infiltration within TME and transforms the phenotype of immune cells into an immune activated state. Hence, after establishing the CAR-Ms platform, we explored a combination strategy involving oAd-CD47 nanoantibody with CAR-Ms for tumor treatment.

oAd-CD47 effectively inhibited the growth of CT26, B16, and ID8 tumor cells (Fig. S4A). Western blot analysis confirmed the successful expression of the anti-CD47 antibody in tumor cells following infection with oAd-CD47 (Fig. S4B). Furthermore, treatment with oAd-CD47 led to a partial blockade of CD47 expression on the surface (Fig. S4C). CT26 tumor cells treated with oAd-CD47 were co-cultured with CAR-Ms to evaluate the phagocytic activity of CAR-Ms (Fig. S5A). The results of FCM analysis showed that the phagocytic activity of CAR-Ms on CT26 tumor cells was significantly improved after oAd-CD47 treatment (Fig. S5B, C).

To determine whether oAd-CD47-treated tumor cells affect the chemotactic behavior of CAR-Ms, a transwell system in which CAR-Ms were co-cultured with CT26 tumor cells was used (Fig. S5D). In this system, the tumor cells treated with oAd-CD47 promoted the migration of CAR-Ms (Fig. S5E). The biodistribution of CAR-Ms in CT26 tumor-bearing mice corroborated these results (Fig. S5F), with the accumulation of CAR-Ms in the tumor of mice intratumorally injected with oAd CD47 increased (Fig. S5G).

### oAd-CD47 and CAR-Ms exert synergistic antitumor efficacy in tumor-bearing mice

**Fig. 5:**
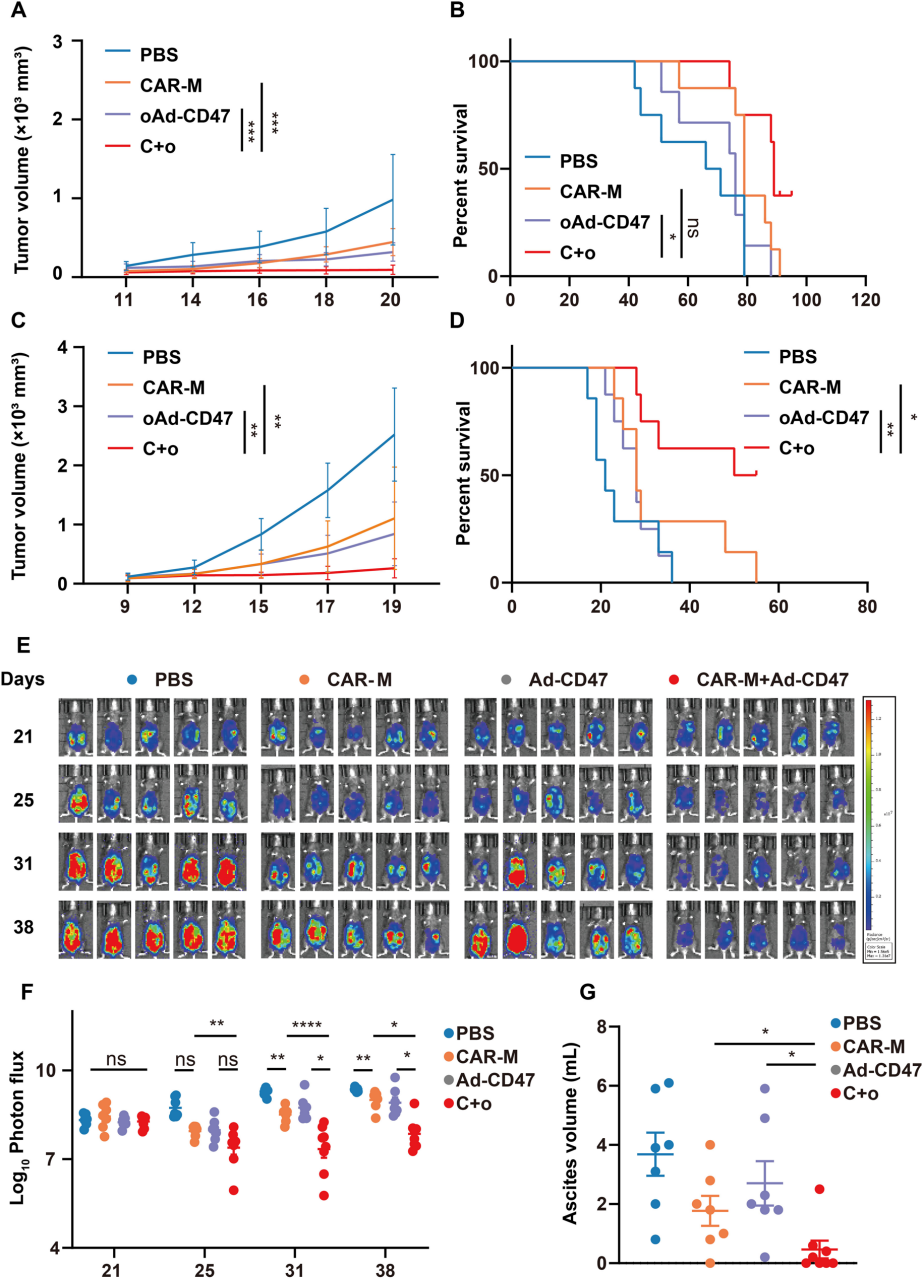
Combination of CAR-Ms and oAd-CD47 primes better anti-tumor activity. **A.** CT26 tumor volume of PBS group, CAR-Ms group, oAd-CD47 group and C + o group measured every two days. **B.** Kaplan-Meier survival curves of mice bearing the CT26 tumor. **C.** B16 tumor volume of different groups measured every two days. **D.** Kaplan-Meier survival curve of mice bearing B16 tumor. **E**. Representative images of ID8-Luc growth in mice after different treatments. (F) Changes in tumor total flux (p/s) in different groups. **(G)** Volume of ascites extracted from mice bearing ID8-luc tumor after different treatments. In all panels, statistical significance was calculated using one-way ANOVA with multiple comparisons, and data are presented as *n* = 6–8. Statistical significance of survival was calculated using the log-rank Mantel-Cox test with df = 2. *P < 0.05, **P < 0.01, ***P < 0.001

The anti-tumor efficacy of CAR-Ms combined with oAd-CD47 (C + o) treatment was evaluated in vivo using two tumor-bearing mice models: CT26 colon cancer subcutaneous tumor model and the B16 melanoma subcutaneous tumor model. After the tumor was established, the mice were randomly assigned into four groups and treated with PBS, CAR-Ms alone, oAd-CD47alone, and CAR-Ms combined with oAd-CD47 (C + o). All three treatment groups, except for PBS, exhibited reduced tumor burden to varying degrees, the C + o treatment group demonstrated superior tumor regression and survival benefits compared to oAd-CD47 group and CAR-Ms group in the CT26 tumor model (Fig. 5A, B). Similar therapeutic effects were observed in the B16 tumor models with low immunogenicity and previously mentioned suboptimal outcomes (Fig. 5C, D).

Ascites accumulation is a hallmark of late-stage ovarian cancer (OC). Ascitic fluid contains diverse cellular components, including cancer cells, immune cells, and a large population of peritoneal macrophages, alongside soluble factors that collectively shape the tumor microenvironment [30]. Based on this characteristic, we evaluated the combined immunotherapy of macrophages and oncolytic virus in an ID8 ovarian cancer ascites model. After 21 days of tumor inoculation, ascites formation was monitored using a bioluminescence imaging system, and treatment administered according the therapeutic schedule (Fig. 5E). The statistical results of bioluminescence signals showed that both CAR-Ms group and oAd-CD47 group had significant tumor reduction to varying degrees after the initial administration, but the tumor size in increased over time. In contrast, the bioluminescence signal in the C + o group remained significantly weaker with no evidence of tumor progression over a two-week period (Fig. 5F). On the 41st day after therapy, we extracted ascites from tumor-bearing mice. Majority of mice in the C + o group did not develop ascites (Fig. 5G), confirming that the combined therapy resulted in a marked therapeutic response.

### oAd-CD47 and CAR-Ms improved phagocytosis of macrophages and reduced inhibitory cells within tumor microenvironment

**Fig. 6:**
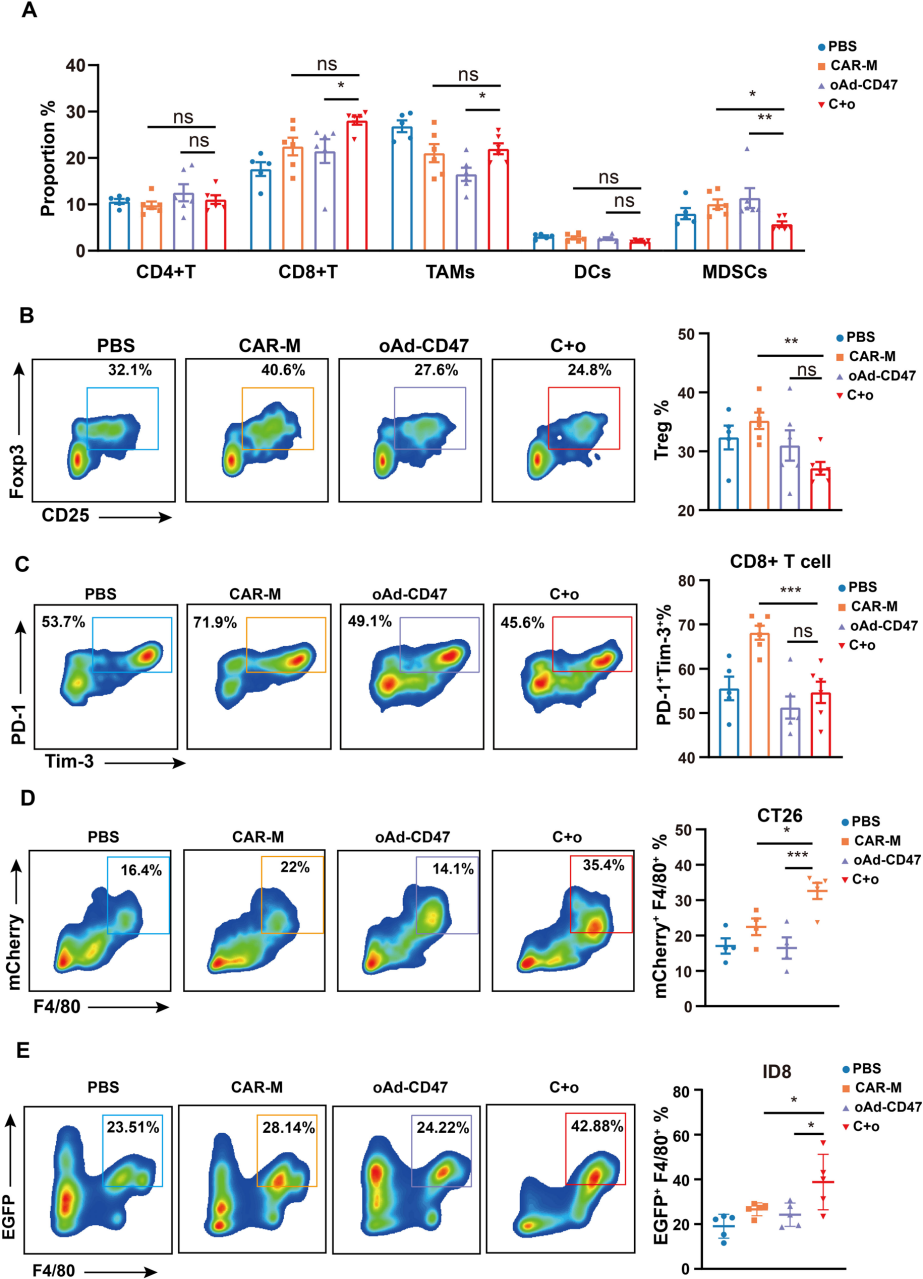
Combination of CAR-Ms and oAd-CD47 results in decreased immunosuppressive cells and enhanced phagocytosis of TAMs in the TME. A. Infiltration of immune cells in the tumor of CT26 model mice via FCM analysis. B. Representative FCM plot of PD-1^+^ Tim-3^+^ in CD8^+^ T cell in the tumor of CT26 model mice. The percentage of PD-1^+^ Tim-3^+^ cells in CD8^+^ T cell. C. Representative FCM plot for Tregs (CD4^+^ CD25^+^ Foxp3^+^) in the tumor of CT26 model mice. The percentage of Tregs. D. Phagocytosis of tumors in the CT26 model mice. E. Phagocytosis in ascites of ID8-EGFP-OVA model mice. In all panels, statistical significance was calculated using one-way ANOVA with multiple comparisons, and data are presented as the mean ± SEM. of n = 5-6 mice. *P < 0.05, **P < 0.01, ***P < 0.001.

The underlying immune mechanisms contributing to the enhanced therapeutic efficacy of the combination therapy were examined. We found that combination therapy did not significantly increase the infiltration of T cells, macrophages, and DCs within the TME compared to CAR-Ms monotherapy (Fig. 6A). However, the accumulation of MDSCs in tumors in C + o group was significantly reduced compared to both CAR-Ms group and oAd-CD47 group (Fig. 6A). Moreover, the population of Tregs in the C + o group was significantly lower compared to the CAR-M group, but not the oAd-CD47 group (Fig. 6C). Analysis of expression of functional markers on T cells revealed that treatment with C + o significantly reduced the number of PD-1^+^Tim-3^+^CD8^+^T cells compared to CAR-M group (Fig. 6B). These findings suggested that the introduction of oAd-CD47 reduced the immunosuppression in TME, offering additional immunological benefits to CAR-Ms. The phagocytic activity of macrophages was significantly enhanced in both the CT26 solid tumor microenvironment (Fig. 6D) and the ID8 OC ascites microenvironment (Fig. 6E) which corroborated our findings. The phagocytic activity of macrophages was significantly enhanced in both CT26 solid tumor microenvironment (Fig. 6D) and ID8 OC ascites microenvironment (Fig. 6E), confirming our findings. By labeling CAR-M in the TME, we found that the proportion of CD206^+^ CAR-M in the C + o group was significantly lower than that in the CAR-M monotherapy group (Fig. S6A). Alternatively, the phagocytosis of CAR-M was significantly upregulated by oAd-CD47 (Fig. S6B).

### The combination of oAd-CD47 and CAR-Ms elicits a multifunctional T cell response

**Fig. 7:**
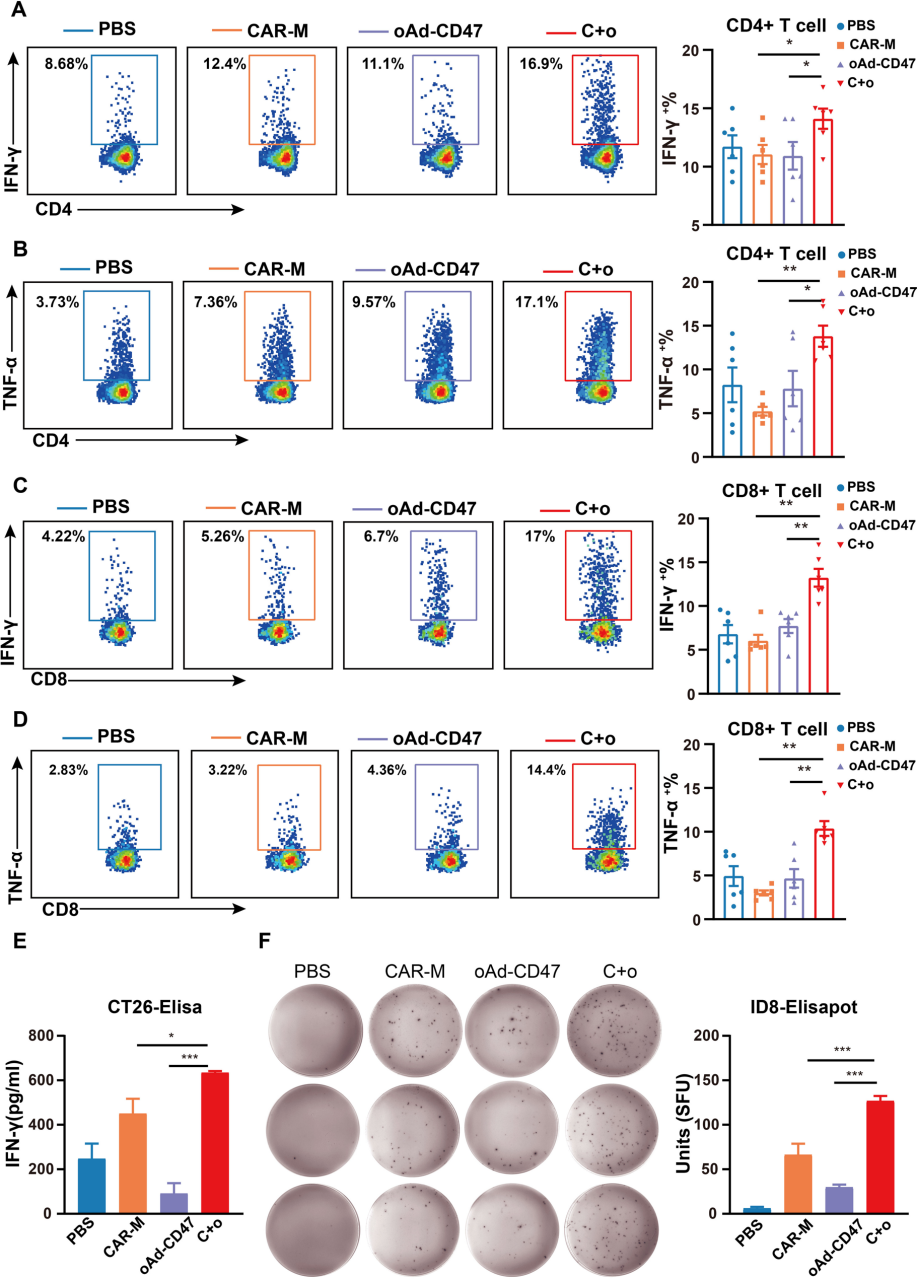
Combination therapy elicits a functional T-cell response in the spleen of the mice. **(A)** Representative FCM plot of IFN-γ^+^ CD4^+^ T cell. The percent of IFN-γ^+^ CD4^+^ T cell. **(B)** Representative FCM plot of TNF-α CD4^+^ T cell. The percent of IFN-γ^+^ CD4^+^ T cell. **C.** Representative FCM plot of IFN-γ^+^ CD8^+^ T cell. The percent of IFN-γ^+^ CD8^+^ T cell. **D.** Representative FCM plot for the TNF-α CD8^+^ T cells. The percent of IFN-γ^+^ CD8^+^ T cell. **E.** IFN-γ secreted by T cells isolated from the spleen of CT26-OVA tumor-bearing mice restimulated in vitro with OVA peptide via ELISA. **F.** Representative IFN-γ elisaspots of T cells isolated from the spleen of ID8-EGFP-OVA tumor-bearing mice restimulated in vitro with OVA peptide. The quantitative data of elisapot. For above panels, statistical significance was calculated using one-way ANOVA with multiple comparisons, and data are presented as the mean ± SEM. of *n* = 5–6 mice. *P < 0.05, **P < 0.01, ***P < 0.001

We conducted analysis on the characteristics of T cells in spleens of mice subjected to combination therapy. The result of FCM analysis indicated that CD4^+^T and CD8^+^T cells in the spleen were functional after treatment with CAR-Ms and oAd-CD47. The proportion of IFN-γ and TNF-α positive cells in CD4 + T and CD8 + T cells was significantly increased (Fig. 7A-D). In combination with the previous findings, we propose that the enhanced phagocytic activity of macrophages in the tumor initiated the antigen-presenting function, leading to a significant increase of antigen-specific T cells, which migrated and eliminated the tumor. We developed a CT26 expressing ovalbumin (CT26-OVA) tumor model to assess whether OVA-specific T cells responses occurred. T cells from the spleen were extracted from CT26-OVA tumor model mice, and their ability to secrete IFN-γ was assessed using enzyme-linked immunosorbent assay. The result showed that the population of spleen cells secreting IFN-γ in the C + o group was significantly increased compared to those in CAR-Ms and oAd-CD47 groups (Fig. 7E). Similar results were demonstrated when the ID8-OVA tumor model was investigated (Fig. 7F).

### The combination of oAd-CD47 and CAR-Ms enhances tumor neoantigen-specific T cell response

**Fig. 8:**
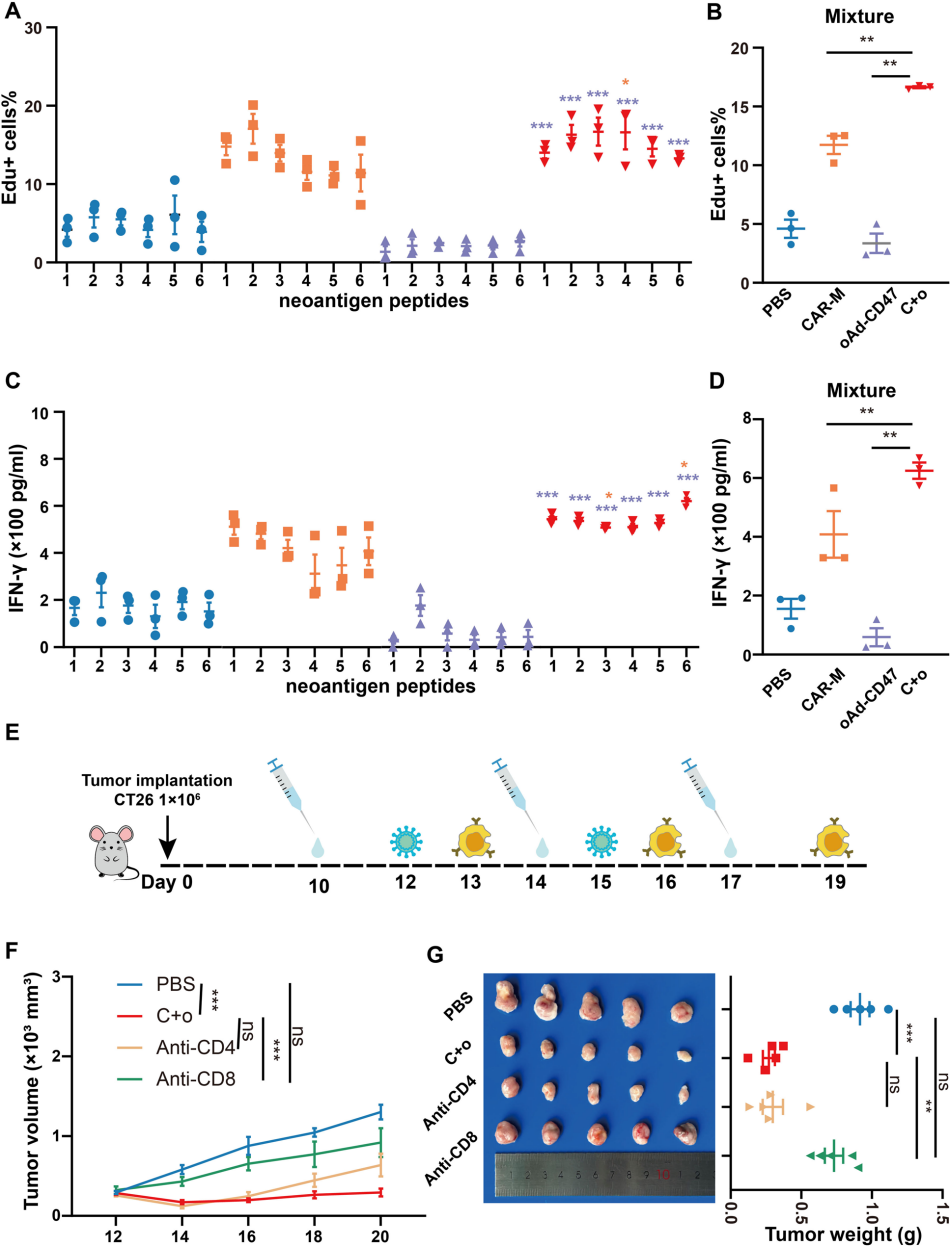
Combination therapy enhanced T cell responses to neoantigen peptides, and the antitumor effect of combination therapy was mainly mediated by CD8^+^T cells. **(A)** The proliferation of T cells after stimulation with a single neoantigen peptide. Yellow * represent CAR-M group versus C + o group, and purple * represent oAd-CD47 group versus C + o group. **(B)** Proliferation of T cells after stimulation with neoantigen peptide mixture. **(C)** The secretion of IFN-γ by T cell stimulated after single neoantigen peptide. Yellow * indicate CAR-M group versus C + o group, and purple * indicate oAd-CD47 group versus C + o group. (D) The secretion of IFN-γ by T cell stimulated with the neoantigen peptide mixture. **(E)** Experimental timeline. The timing of injection of anti-CD4/anti-CD8, oAd-CD47 and CAR-Ms into mice bearing CT26 tumor. **(F)** Tumor volume of different groups. **(G)** Representative tumors for different groups. The tumor weight of differential group. Statistical significance was calculated with one-way ANOVA with multiple comparisons, and data are presented as the mean ± SEM. *P < 0.05, **P < 0.01, ***P < 0.001

To investigate whether the combination therapy facilitates the induction of tumor antigen-specific T cells with antitumor activity comparable to OVA-specific T cells, we examined its effects on neoantigen-specific T cell responses. Recently, D’Alise et al. used whole exome and transcriptome sequencing analysis combined with mass spectrometry to identify neoantigens in CT26 tumor cells [27]. Using their findings, six mutant neoantigen epitopes were synthesized for use in this study. To validate the occurrence of neoantigen-specific T cell response, splenic T lymphocytes were extracted from mice with CT26 tumor model and cultured in pools of six neoantigen peptides and as well as mixed peptides. T cells from mice treated with CAR-Ms responded to the stimulation of the six neoantigen peptides and displayed significant T cell proliferation and IFN-γ secretion compared to mice treated with PBS (Fig. 8A, C). Administration of oAd-CD47 alone did not significantly induce a neoantigen-specific T cell response in tumor-bearing mice. However, T cells from mice treated with C + o responded most vigorously to the stimulation of the six neoantigen peptides compared to mice treated with CAR-Ms and oAd-CD47 alone, demonstrating T cell proliferation and IFN-γ secretion. (Fig. 8B, D). These findings support that CAR-Ms combined with oAd-CD47 is more effective in activating neoantigen-specific T cell responses.

These findings suggest that T cells are involved in anti-tumor effects mediated by the CAR-Ms and oAd-CD47 combination therapy. To identify the specific T cell subset that play a major role in the anti-tumor activity, we performed depletion studies using anti-CD4 and anti-CD8 antibodies to selectively deplete CD4^+^ T and CD8^+^ T cells, respectively (Fig. 8E). The depletion effect was verified using FCM analysis (Fig. S7). As shown in Fig. 8F and G, the depletion of CD8^+^T cells significantly impaired the anti-tumor efficacy of the combination therapy, whereas CD4 T cells depletion exerted no significant effect on the therapeutic effect of CAR-Ms and oAd-CD47.

## Discussion

Studies have demonstrated that TAMs are an important component of the TME, involved in the regulation of angiogenesis, coordination of cancer cell proliferation, metastasis, and immunosuppression, and occurrence of resistance to chemotherapeutic drugs and checkpoint blocking immunotherapy [31]. Macrophages exhibit phenotypic plasticity, and when appropriately activated, they can mediate phagocytosis of cancer cells and induce cytotoxic tumor killing. This connection between innate immunity and adaptive anti-tumor immunity makes macrophages attractive targets for cancer therapy. Previous clinical trials investigated two strategies for macrophage elimination and inhibiting macrophage recruitment (NCT00588913, NCT04123379, NCT02826486) [32]. In future, reprogramming or repolarizing TAM to produce anti-tumor proinflammatory effects is expected to be an important macrophage targeting strategy. Kang et al. used adenovirus vector Ad5F35 to deliver HER2-targeting chimeric antigen receptors to macrophages to establish proinflammatory CAR macrophages [17]. Elsewhere, Mikyung et al. stimulated TAMs to differentiate into CAR-M1 macrophages in vivo using nanocomplexes of macrophage-targeted nanocarriers and CAR IFN-γ-coding plasmid DNA, which circumvented the limitations of CAR-T therapy in the treatment of solid tumor [33]. Previous research has underscored the significance of repolarizing TAMs to M1 macrophages in CAR-mediated tumor phagocytosis, anti-tumor immunoregulation, and solid tumor growth inhibition. Building upon these findings, we developed CAR-Ms designed to induce M1 polarization by employing adenovirus vectors expressing PD-L1 targeting CAR and IFN-γ genes. Our results showed that the percentage of cells with M1-type marker positive (iNOS^+^) in BMDM modified with Ad-CAM was 43.50%. Moreover, the results demonstrated that CAR-Ms enhanced anti-tumor immunity by decreasing the number of M2 macrophages in TME to increase the proportion of M1 macrophages and improve the antigen-presenting capacity of macrophages and DCs in TME. These results demonstrated that the anti-tumor efficacy and therapeutic mechanisms of CAR macrophages and adaptive anti-tumor immunity were comparable to those reported in previous studies.

As a target with broad spectrum applicability, PD-L1 is highly expressed in a variety of solid tumors (such as lung cancer, melanoma, triple-negative breast cancer) and hematological tumors (such as Hodgkin’s lymphoma) [34]. CAR-M targeting PD-L1 not only directly reduced tumor burden by selectively eliminating PD-L1⁺ tumor cells, but also alleviated PD-1/PD-L1-mediated T cell depletion. Therefore, we introduced the IFN-γ gene into the CAR-M to enhance its anti-tumor function by activating the proinflammatory polarization of macrophages. However, PD-L1 is not only expressed on the surface of tumors, which is involved in immune escape, but also expressed on the surface of antigen-presenting cells (DC cells, macrophages, etc.) and vascular endothelial cells under the stimulation of IFN-γ [35]. Therefore, an important question is whether PD-L1-targeting CAR-M engulfs other immune cells in addition to tumor cells or even CAR-M itself. In a study employing genetically engineered high-affinity natural killer (haNK) cells, PD-L1-targeted CAR haNKs effectively inhibited tumor growth in a PD-L1-dependent manner and concurrently depleted myeloid cell populations that endogenously express high levels of PD-L1 [36]. In addition, the results of another study also demonstrated the cytotoxic effect of PD-L1-CAR T cells on non-malignant cells [37]. Although there have been no reports of PD-L1-targeting CAR-M, this phenomenon prompted us to investigate the cytotoxic effects of PD-L1-overexpressing nontumor cells. The results showed that BMDM from which CAR-M was derived exhibited high expression PD-L1 (Fig. S8A). Therefore, we incubated CFSE-labeled BMDM with DiD-labeled CAR-M, while co-incubated BMDM labeled with both dyes served as a control. The results showed that the proportion of DiD cells under the CSFE^+^ gate in the CAR-M group was only 5.8%, which was not statistically different from that in the BMDM group (Fig. S8B). We propose that the active inhibition of self-recognition signals present in the body results in the protection of normal cells by CAR-M. The immune system recognizes outsiders by identifying determinants or “self-markers” that are absent on host cells. Studies have shown that CD47 can act as a self-marker on mouse red blood cells to prevent this elimination by binding to the inhibitory receptor SIRPα [38]. Similarly, macrophages also use many of these non-specific activating receptors, such as Sialic Acid Binding Ig Like Lectin 10 (siglec-10), and rely on the presence or absence of differentiation cluster 24 (CD24) to distinguish self from foreign cells [39]. CD24 is normally expressed on immune cells and epithelial cells, which leads to inhibition of macrophage-mediated phagocytosis [40]. Specific, whether non-tumor cells with high PD-L1 expression are protected by this mechanism needs to be further investigated.

Although CAR-Ms have demonstrated good efficacy in several mice models with strong proinflammatory characteristics in the TME, we must consider the complexity of different tumor models as well as the actual tumor microenvironment in humans [17]. Despite our efforts, CAR-Ms did not demonstrate satisfactory therapeutic efficacy in low immunogenic mouse tumor models. The high heterogeneity of tumor cells suggests that targeting a single antigen may not be sufficient to induce robust CAR-mediated antitumor effect [17]. Additionally, tumor cells can express “don’t eat me” signaling proteins on the cell membrane, such as CD47 and CD24, to avoid macrophage phagocytosis and thus escape immune response. Moreover, the suppression of CAR-Ms by the inhibitory immune cells (MDSCs, Tregs) and the immunodepleted T cells lead to the impairment of adaptive anti-tumor immunity. In addition to the TME, the limited survival time and the ability of CAR macrophages to function continuously in vivo may influence the durability of therapeutic effects^37^. Therefore, further investigation of combination therapies involving CAR-Ms is essential.

Currently, the combination of CAR-Ms with other therapies is in the process of development [41]. For example, there is a potential synergistic antitumor effect in combination with checkpoint inhibitors [42]. According to a study, the combination of CAR-M with PD-1 checkpoint inhibitor therapy has synergistic antitumor effect in the CT26-HER2 mouse model, which provided rationale for the combination of CAR-Ms with immune checkpoint inhibitors [43]. Enhancing the phagocytic capabilities of macrophages by targeting inhibitory checkpoints within the innate immune system presents a unique avenue for combination therapies with CAR-Ms. Yang et al. revealed that treatment of mice with LNP producing CAR-Ms and CD24-Siglec-G blockers significantly improved the phagocytosis of liver macrophages and effectively inhibited tumor growth [44]. In the current research on phagocytosis checkpoints, CD47 has been extensively investigated [45–47]. Monoclonal antibodies against CD47 not only enhance macrophage phagocytosis, but also effectively improved adaptive anti-tumor immunity mediated by macrophages [45]. In this study, we tested the combination of CAR-Ms and oncolytic adenovirus expressing anti-CD47 antibody (oAd-CD47). We administered oAd-CD47 intratumorally, aligning with the established clinical practice of delivering oncolytic adenoviruses directly to the tumor site. This approach was chosen to minimize potential off-target effects associated with systemic administration of anti-CD47 antibodies [24]. Our results demonstrated the best antitumor efficacy of CAR-Ms could be enhanced by oAd-CD47. Furthermore, C + o induced higher macrophage phagocytosis in the TME compared with CAR-Ms and oAd-CD47 treatments alone. Oncolytic adenovirus killed the tumor and remodeled the immunosuppressed TME to establish a pro-inflammatory state. Moreover, oAd-CD47 significantly decreased the number of MDSCs and Tregs in the TME and effectively reversing the increased expression of PD-1 and Tim-3 on CD8^+^T cells, thereby enhancing anti-tumor T cell responses.

Studies have demonstrated that tumors exhibit high genetic heterogeneity and individual tumors with numerous genetic mutations express several potentially immunogenic neoantigens [48]. Identification of such tumor-specific mutations in an individual patient can enhance effective antitumor specific T-cell response and improve efficacy of cancer therapies, including immunotherapy [49]. Personalized therapeutic cancer vaccine regimens based on neoantigens are currently being evaluated in clinical trials for patients with melanoma (NCT00683670) and glioblastoma (NCT02149225). While still in the early stages of development, these vaccine approaches have demonstrated promising safety profiles and therapeutic potential [49]. Oncolytic viruses promote neoantigen presentation and activate systemic T-cell responses against dominant and subdominant neoantigens [50]. In addition, adenoviruses are the preferred platforms for neoantigen-based immunotherapy [27, 51]. However, oAd-CD47 treatment alone did not induce T cell proliferation or IFN-γ secretion following neoantigen peptide stimulation. This may be attributed to two factors: (1) oAd-CD47 lacked a payload that directly activated T cells; (2) Insufficient neoantigen presentation may have limited its ability to elicit T cell responses. In contrast, based on our results, CAR-Ms monotherapy promoted the antigen presentation ability of APCs and response to the stimulation of some neoantigen peptides. Compared with CAR-Ms monotherapy, C + o group significantly induced stronger neoantigen-specific T cell response, suggesting that the role of oAd-CD47 in this process.

Dendritic cells (DCs) are widely recognized as key players in initiating and maintaining antitumor T cell immunity. One strategy to induce neoantigen-specific immune responses involves isolating DCs from patients, exposing them to tumor lysates in vitro, and reinfusing them into the patients to stimulate immune responses against tumor-associated antigens or neoantigens [52]. Evidence from previous studies has provided supported the role of macrophages in neoantigen-specific T cell immunity. In MHC class I-deficient cancers, CD8^+^T cells recognize neoantigens cross-presented by interleukin2-activated macrophages in the TME [53]. Furthermore, given our previous findings that CAR-Ms can activate both macrophages and dendritic cells, the precise mechanisms underlying neoantigen-specific recognition in combination therapies warrant further investigation.

Overall, our results indicated that PD-L1-targeted CAR-Ms displayed a predominantly proinflammatory M1 phenotype and exerted antitumor effects in vitro and in vivo. These findings support a therapeutic strategy to enhance anti-tumor immunity by combining CAR-Ms with oAd-CD47. We speculate that this combination may leverage the potential synergistic actions of CAR-M phagocytosis/proinflammatory activity, oAd-mediated immunogenic oncolysis, and CD47-SIRPα blockade to promote neoantigen-specific T cell responses. Considering the clinical limitations of current CAR-T cell therapy in the treatment of solid cancers, the combination strategy of CAR-M cell therapy with oAd-CD47 may be an effective approach for personalized tumor-specific oncolytic immunotherapy.

## Conclusion

In conclusion, our findings suggest that the combination of CAR-Ms and oAd-CD47 exhibits potentially synergistic anti-tumor activity in the tested preclinical models. This work provides initial evidence supporting the feasibility and rationale for exploring CAR-Ms in combination with other immunotherapeutic agents, such as oncolytic viruses. However, considering limitations such as the inherent differences between mouse models and human tumors and the potential off-target effects that have not been fully characterized, the translational potential of this specific combination requires further validation in more complex models and ultimately in clinical settings.

## Supplementary Information

Below is the link to the electronic supplementary material.

Supplementary Material 1

## Data Availability

No datasets were generated or analysed during the current study.

## Abbreviations

CAR: Chimeric antigen receptor
CAR-Ms: Chimeric antigen receptor macrophages
TAMs: Tumor-associated macrophages
TSA: Tumor-specific antigen
Tregs: Regulatory T cells
MDSCs: Myeloid suppressive cells
PD-1: Programmed cell death 1 receptor
PD-L1: programmed cell death ligand 1
IFN-γ: Interferon-gamma
TME: Tumor microenvironment
APC: Antigen-presenting cell
DCs: Dendritic cells
