# Supplementary material for: CD47 antibody-armed oncolytic adenovirus promotes chimeric antigen receptor macrophage phagocytosis and antitumor immunity
Source: Exp Hematol Oncol. 2025 Aug 14;14:106. doi: 10.1186/s40164-025-00696-7 (PMC12355751; doi:10.1186/s40164-025-00696-7)
Supplement: Supplementary file 1 — Supplementary Material 1 [file 40164_2025_696_MOESM1_ESM.docx]

**CD47 antibody-armed oncolytic adenovirus promotes chimeric antigen receptor macrophage phagocytosis and antitumor immunity**

**Zhongbing Qi^1#^, Shichuan Hu^1#^, Jing Zhao^2^, Xianglin Xu^1^, Anliang Huang^4^, Yu Qin^1^, Yao Zhang^1^, Qingzhe Yang^1^, Jianchuan Hu^1^, Chao Su^3^ and Ping Cheng^1^*.**

^1^State Key Laboratory of Biotherapy and Cancer Center/Collaborative Innovation Center for Biotherapy, West China Hospital, Sichuan University, 17 People’s South Road, Chengdu, 610041, PR China.

^2^ Department of Biotherapy, Cancer Center, West China Hospital of Sichuan University, 17 People’s South Road, Chengdu, 610041, PR China.

^3^Department of Biotherapy, Cancer Center, West China Hospital, Laboratory of Integrative Medicine, Clinical Research Center for Breast and State Key Laboratory of Biotherapy, Sichuan University, Chengdu, Sichuan 610041, China.

^4^Department of Pathology, Chengdu Fifth People’s Hospital, Chengdu 610041, PR China.

^#^ These authors contributed equally to this work.

*Corresponding author: Prof. Ping Cheng, Department of Biotherapy, Cancer Center and State Key Laboratory of Biotherapy, West China Hospital, Sichuan University, Chengdu, 610041, China. Email: [chengping@scu.edu.cn](mailto:chengping@scu.edu.cn); [ping.cheng@foxmail.com](mailto:ping.cheng@foxmail.com).


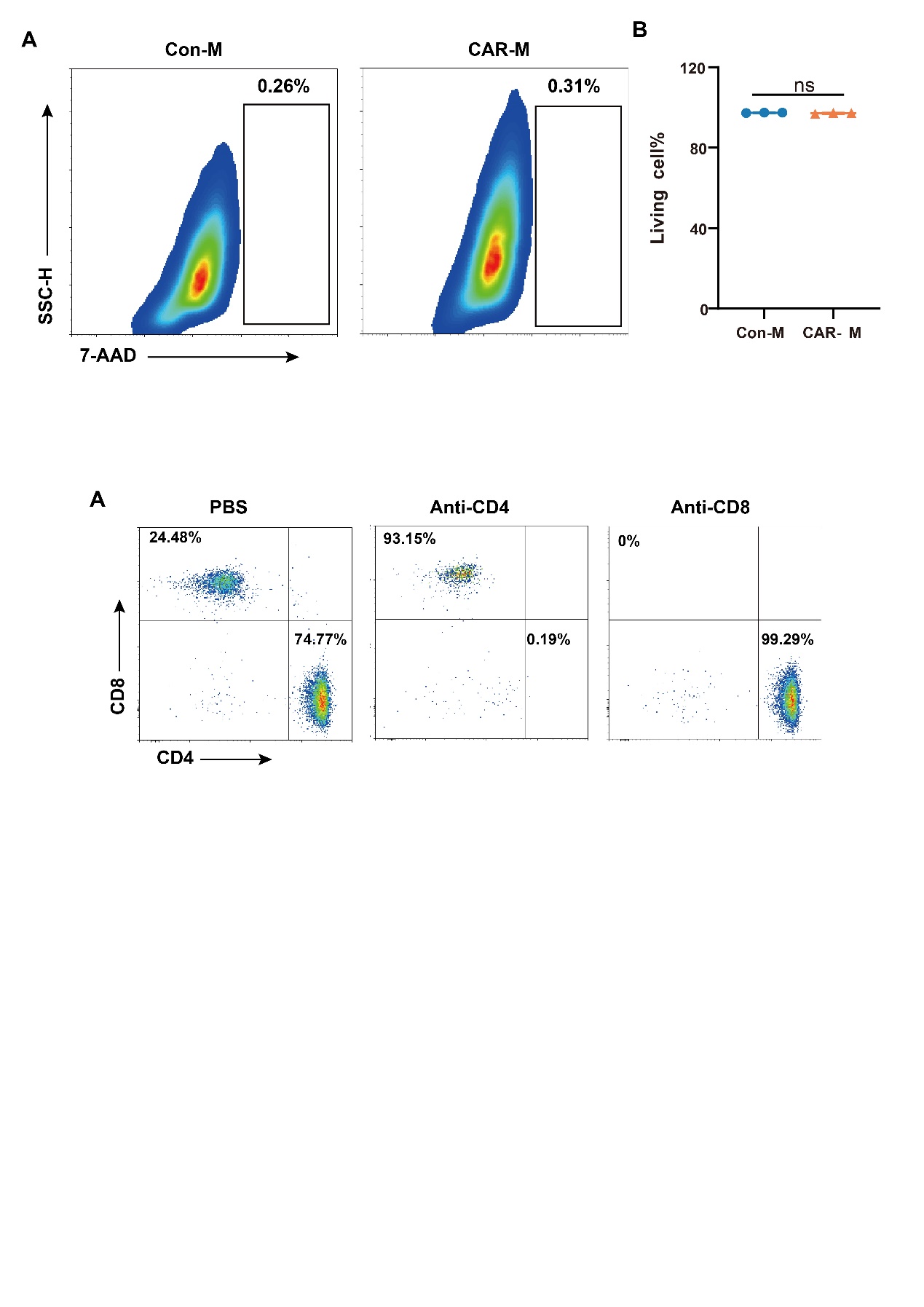
 **Figure S1. Adenovirus infection does not cause macrophages death.**

7 - AAD dyeing is used to distinguish dead and live cell. **A.** Representative FCM plot of 7-AAD^+^ cells. **B.** Statistical histogram of living cells. Data represent the mean ± SEM of n = 3 and statistical significance was calculated using unpaired t-test.


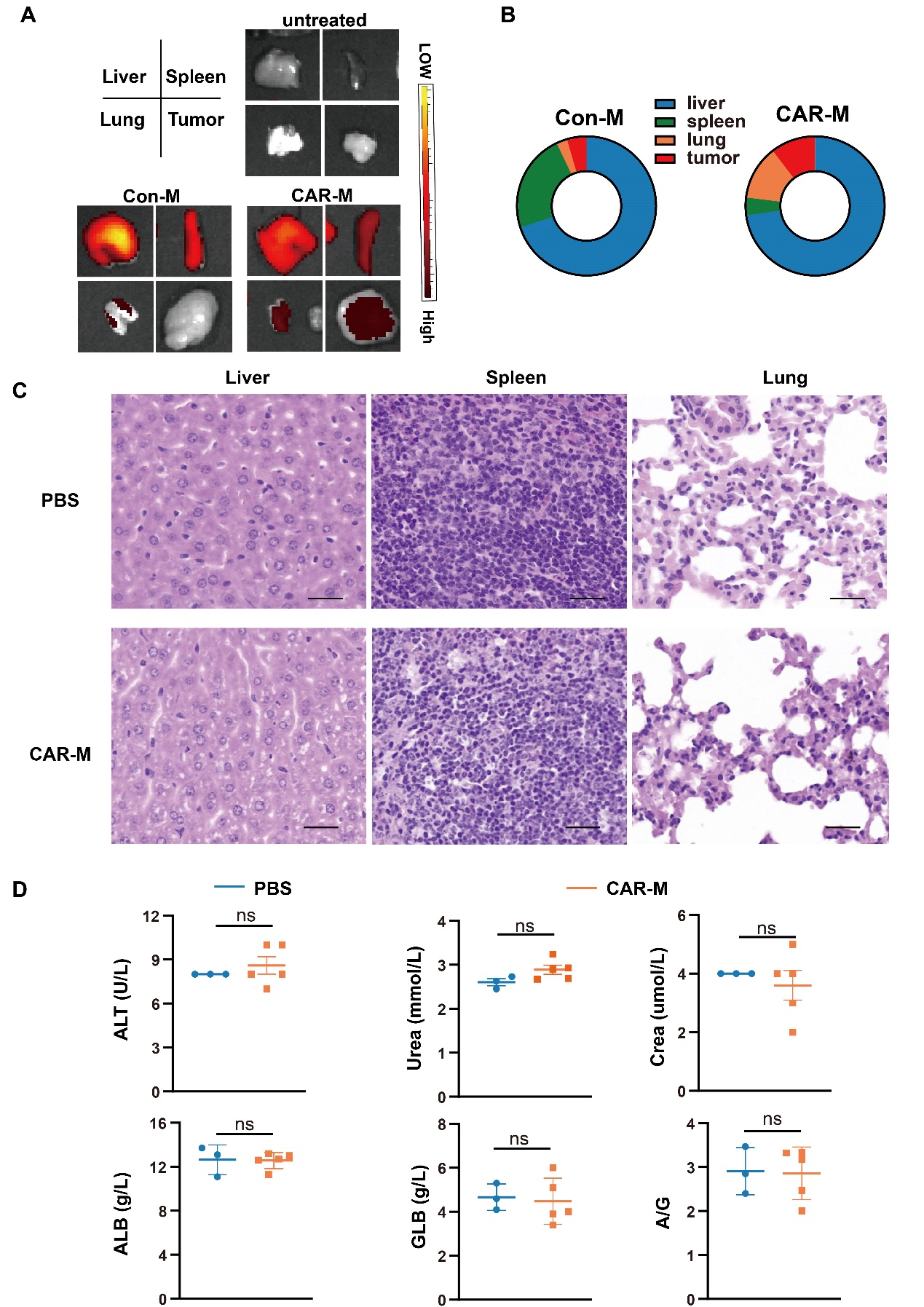


**Figure S2. Biodistribution and safety of CAR-Ms in mice.**

**A.** Representative ex vivo fluorescence images of major organs and tumor tissues 48 h after the last injection of CAR-Ms or Con-Ms. **B.** Pie chart of CAR-Ms’ or Con-Ms’ distribution from one of the mice in the CAR-M group and CAR-M groups. **C.** Representative images of hematoxylin and eosin-stained liver, spleen and lung tissues from PBS group and CAR-M group mice. Scale bars = 50 μm. **D.** Expression of alanine transaminase (ALT), blood urea nitrogen (BUN), creatinine (Cr), albumin (ALB), globulin (GLB) and Ratio of ALB and GLB (A/G) in peripheral blood of mice serum. Data represent the mean ± SEM of n = 3-5 mice and statistical significance was calculated using unpaired t-test. ns has no significant P value.


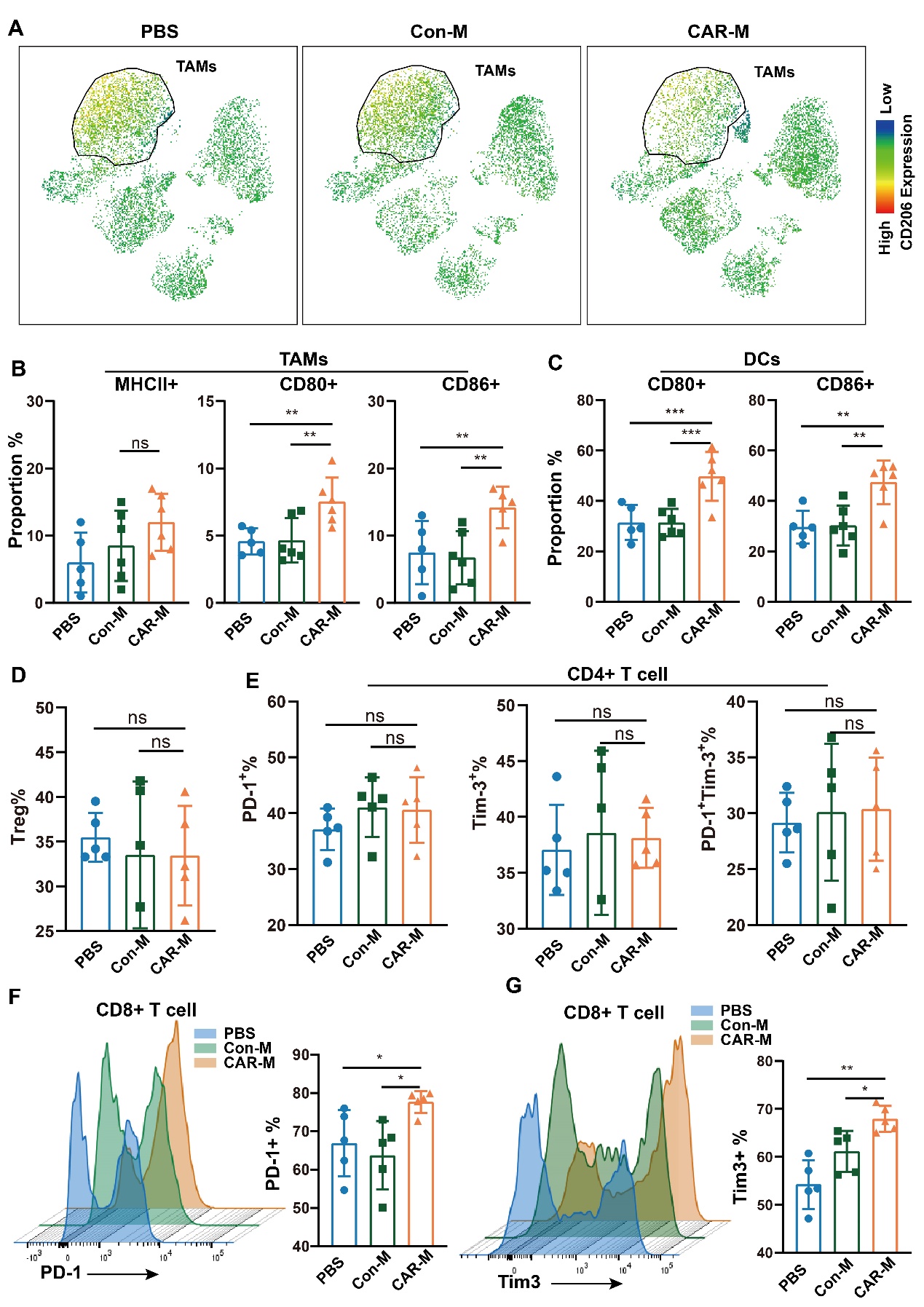


**Figure S3. Changes of some immune cells in the TME of CT26 tumor-bearing mice after treated with PBS, Con-Ms and CAR-Ms.**

**A.** Heat map of CD206 expression on TAMs. **B.** Percent of MHCII^+^, CD80^+^, CD86^+^ cells in TAMs. **C.** Percent of CD80^+^, CD86^+^ cells in DCs. **D.** Percent of Tregs in TME. **E.** Percent of PD-1^+^, Tim3^+^, and PD-1^+^Tim3^+^ cells in CD4^+^T.

**F.** Representative FCM plot of PD-1^+^CD8^+^T cell. And Percent of PD-1^+^ cells in CD8^+^T cell. **G.** Representative FCM plot of Tim3^+^CD8^+^T cell. And Percent of Tim3^+^ cells in CD8+T cell. For all panels, statistical significance was calculated with one-way ANOVA with multiple comparisons, and data represent the mean ±SEM. of n = 5 mice. *P < 0.05, **P < 0.01, ***P < 0.001.


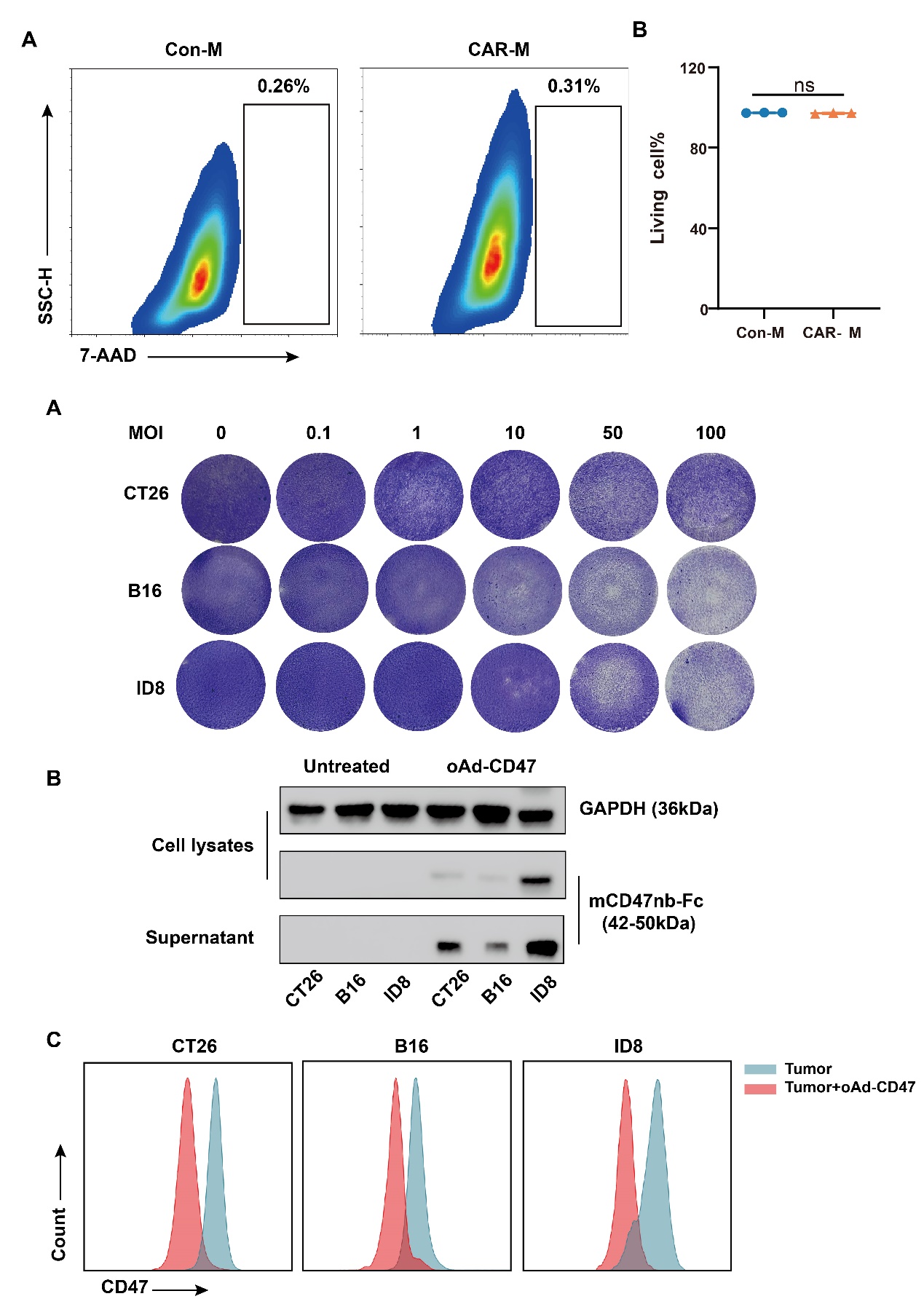


**Figure S4 The cytotoxic effect of oAd-CD47 on tumor cells and its inhibitory effect on CD47 expression on the surface of tumor cells.**

A. Representative images of tumor cells infected with oAd-CD47 at different MOI values for 48 hours and stained with crystal violet. B. Quantitative expression of mCD47nb-Fc in different tumor cells at 48 h after infection with oAd-CD47 at a dose of MOI 25. C. The CD47-blocking function of oAd-CD47 on tumor cells.


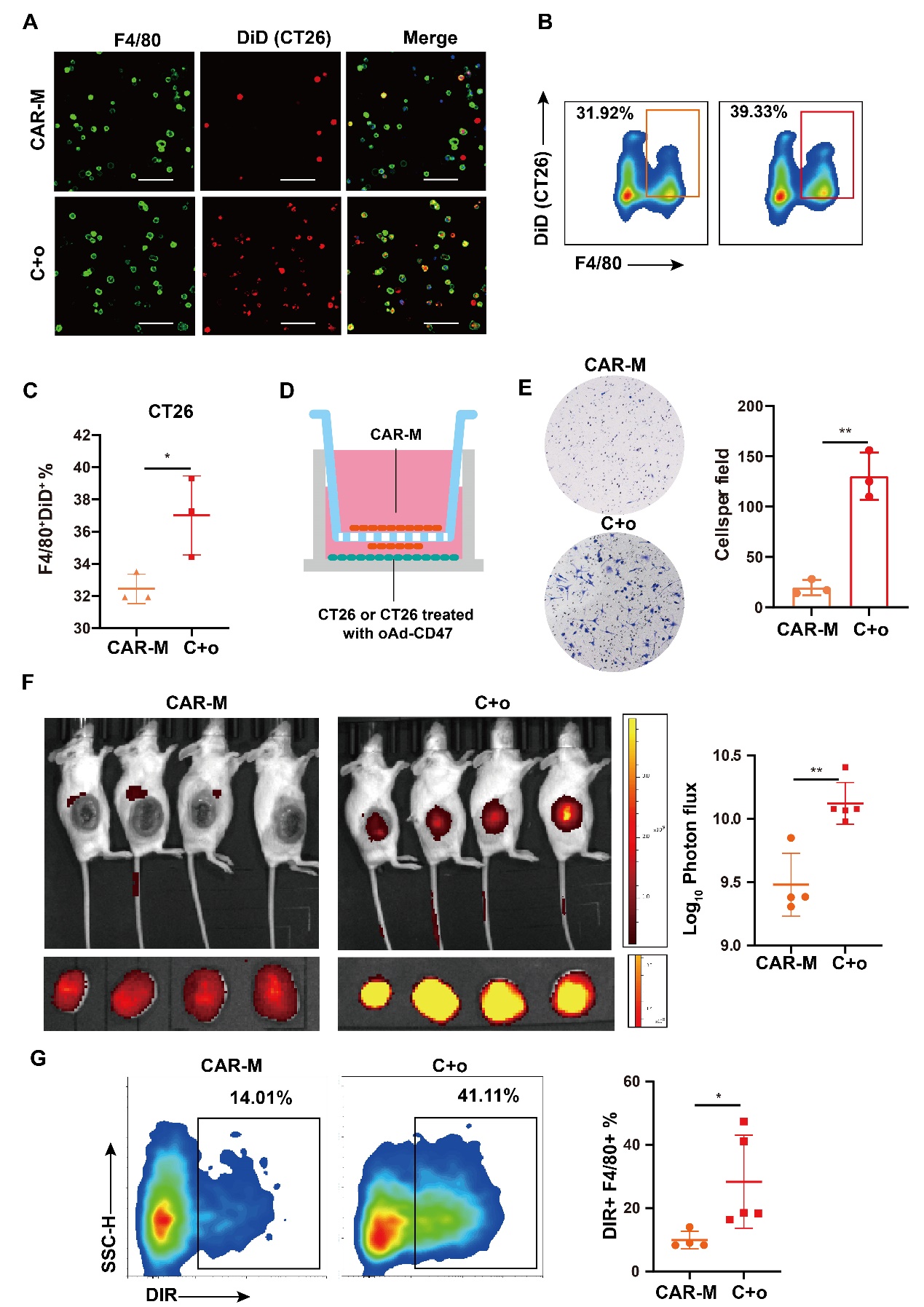


**Figure S5. CAR-Ms showed a stronger phagocytosis on tumor cells infected with oAd-CD47, and a tropism migration towards them.**

**A.** Phagocytosis by confocal microscopy. Scale bars = 50 μm. **B.** Representative FCM plot of phagocytosis. **C.** Statistical histogram of phagocytosis. Statistical significance was calculated with one-way ANOVA with multiple comparisons, and data represent n = 3 technical replicates. **D.** In the co-culture system of CAR-Ms and tumor cells, CT26 cells seeded in 24-well plates attracted CAR-Ms placed in the upper chambers of a Transwell system to migrate across the membrane. Following the removal of cells from the inner side of the Transwell insert, the cells that had adhered to the outer surface of the membrane were stained with crystal violet and subsequently quantified. **E.** Representative microscopic picture of the outer surface of the transwell membrane stained with crystal violet. And quantification of CAR-M cell numbers in each field. The cell counts were obtained from at least three random fields and the data represent the mean ± s.e.m. Statistical significance was calculated using unpaired t-test. **F.** CT26 tumor-bearing mice received three intravenous injections of DiR-labeled Con-Ms or CAR-Ms (2×10⁶ cells per injection, with a three-day interval between each injection) and two intratumoral injections of PBS or oAd-CD47 (2×10⁸ PFU per injection). Mice were sacrificed 5 days after the last injection, and tumors were harvested for *in vitro* imaging. Accumulation of DIR - labeled CAR-Ms in tumor of CAR-M group and C+o group. And statistical histogram of total flux (p/s) in different groups. Data analysis was performed using unpaired t-test and data represent the mean ± s.e.m. of (n=4 mice). **G.** Representative FCM plot of accumulation of DIR - labeled CAR-Ms in tumor tissues. And percent of DIR^+^ cells in F4/80^+^ cells of tumor. Data analysis was performed using unpaired t-test and data represent the mean ± SEM of (n=4 mice). For all panels, *P < 0.05, **P < 0.01, ***P < 0.001.


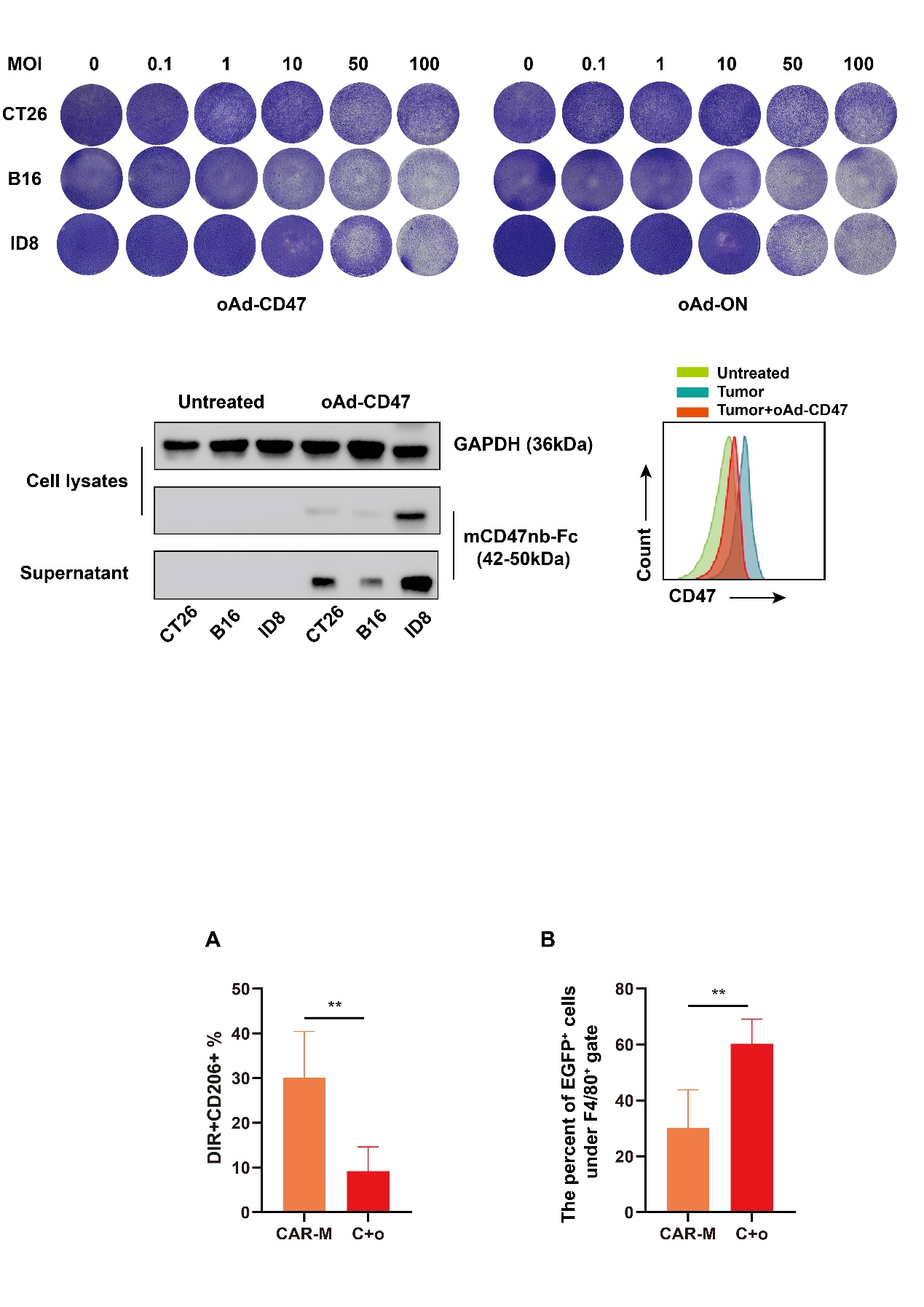


**Figure S6. CAR - M Typing and Phagocytosis within the TME**

To explore the characteristics of CAR – Ms in combination therapy, DiR (a fat - soluble near-infrared fluorescent dye)-labeled CAR-Ms were injected into ID8-EGFP ovarian cancer peritoneal metastasis mice. A. Proportion of CD206⁺ DiR⁺ Cells detected by FCM. B. Proportion of EGFP⁺ Cells within the F4/80⁺ Gate, counted from within the DiR⁺ gate detected by FCM. Data analysis was performed using unpaired t-test and expressed the mean ± SEM of (n=5 mice). For all panels, *P < 0.05, **P < 0.01, ***P < 0.001.


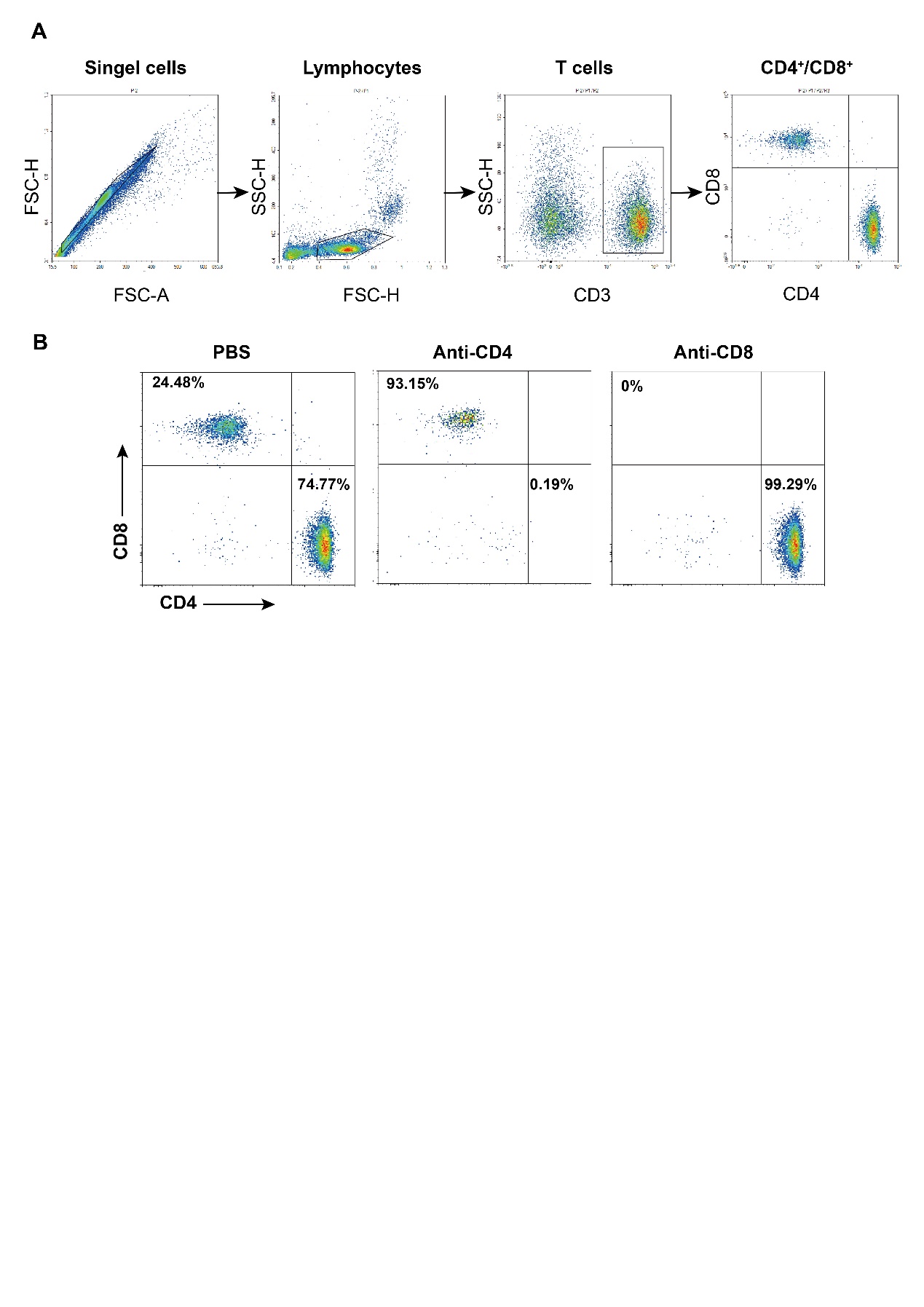


**Figure S7: Flow Cytometric Analysis of Circulating T-Cell Subsets in Mice Treated with Anti-CD4/CD8 Antibodies.**

Mice bearing CT26 subcutaneous tumor received intraperitoneal injection of 200 μg anti-CD4 or anti-CD8 on day 10, 14 and 17. Forty-eight hours after the initial administration of CD4 and CD8 antibodies, blood samples were collected from the mice. These samples were then subjected to flow cytometry to analyze the T cell populations. Panel A displays the gating strategy diagram used for T cell identification. B. Representative Flow Plots of CD4⁺ and CD8⁺ T Cells.


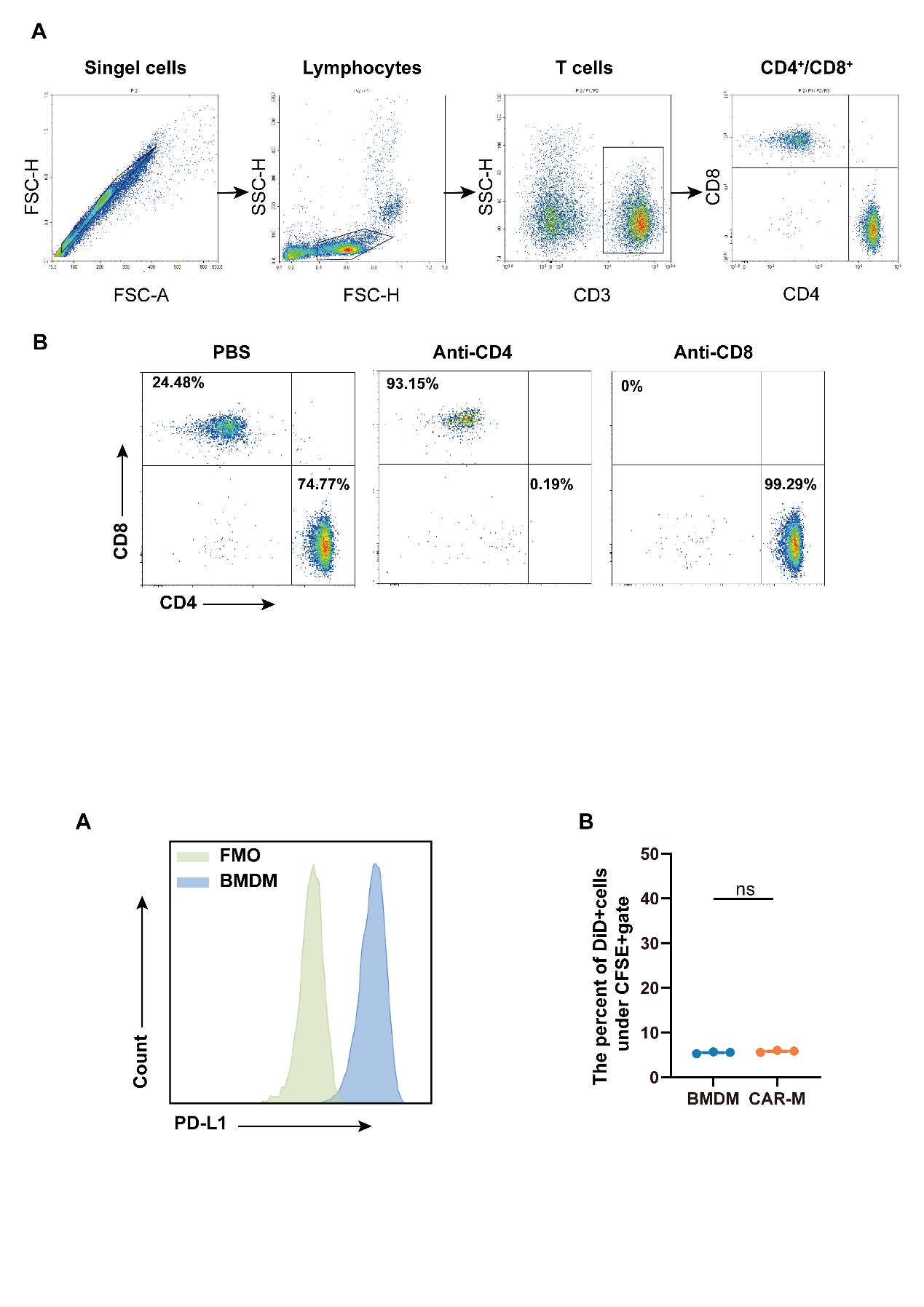


**Figure S8: Phagocytosis between BMDM and CAR-M.**

A. The expression on BMDMs. B. Proportion of CSFE^+^ DiD^+^ cells in the co-incubation experiment between BMDM and CAR-M as determined by FCM. Statistical analysis was performed using unpaired t-test and data represent the mean ± SEM of (n=3). For all panels, *P < 0.05, **P < 0.01, ***P < 0.001.
